# Identification and functional analysis of glycemic trait loci in the China Health and Nutrition Survey

**DOI:** 10.1371/journal.pgen.1007275

**Published:** 2018-04-05

**Authors:** Cassandra N. Spracklen, Jinxiu Shi, Swarooparani Vadlamudi, Ying Wu, Meng Zou, Chelsea K. Raulerson, James P. Davis, Monica Zeynalzadeh, Kayla Jackson, Wentao Yuan, Haifeng Wang, Weihua Shou, Ying Wang, Jingchun Luo, Leslie A. Lange, Ethan M. Lange, Barry M. Popkin, Penny Gordon-Larsen, Shufa Du, Wei Huang, Karen L. Mohlke

**Affiliations:** 1 Department of Genetics, University of North Carolina at Chapel Hill, Chapel Hill, North Carolina, United States of America; 2 Department of Genetics, Shanghai-MOST Key Laboratory of Heath and Disease Genomics, Chinese National Human Genome Center and Shanghai Industrial Technology Institute, Shanghai, China; 3 Lineberger Comprehensive Cancer Center, School of Medicine, University of North Carolina at Chapel Hill, Chapel Hill, North Carolina, United States of America; 4 Department of Medicine, University of Colorado Anschutz Medical Campus, Aurora, Colorado, United States of America; 5 Department of Nutrition, University of North Carolina at Chapel Hill, Chapel Hill, North Carolina, United States of America; 6 Carolina Population Center, University of North Carolina at Chapel Hill, Chapel Hill, North Carolina, United States of America; Massachusetts General Hospital, UNITED STATES

## Abstract

To identify genetic contributions to type 2 diabetes (T2D) and related glycemic traits (fasting glucose, fasting insulin, and HbA1c), we conducted genome-wide association analyses (GWAS) in up to 7,178 Chinese subjects from nine provinces in the China Health and Nutrition Survey (CHNS). We examined patterns of population structure within CHNS and found that allele frequencies differed across provinces, consistent with genetic drift and population substructure. We further validated 32 previously described T2D- and glycemic trait-loci, including *G6PC2* and *SIX3-SIX2* associated with fasting glucose. At *G6PC2*, we replicated a known fasting glucose-associated variant (rs34177044) and identified a second signal (rs2232326), a low-frequency (4%), probably damaging missense variant (S324P). A variant within the lead fasting glucose-associated signal at *SIX3-SIX2* co-localized with pancreatic islet expression quantitative trait loci (eQTL) for *SIX3*, *SIX2*, and three noncoding transcripts. To identify variants functionally responsible for the fasting glucose association at *SIX3-SIX2*, we tested five candidate variants for allelic differences in regulatory function. The rs12712928-C allele, associated with higher fasting glucose and lower transcript expression level, showed lower transcriptional activity in reporter assays and increased binding to GABP compared to the rs12712928-G, suggesting that rs12712928-C contributes to elevated fasting glucose levels by disrupting an islet enhancer, resulting in reduced gene expression. Taken together, these analyses identified multiple loci associated with glycemic traits across China, and suggest a regulatory mechanism at the *SIX3-SIX2* fasting glucose GWAS locus.

## Author summary

Type 2 diabetes risk and levels of glucose, insulin, and HbA1c are heritable traits correlated with risk of other diseases and mortality. To identify genetic variants associated with these traits, we studied up to 7,178 men and women from nine provinces in China. We found established variants that could affect fasting glucose located in or near genes named *G6PC2* and *SIX3*. One of the variants at *G6PC2* changes the protein sequence and is predicted to affect how the protein functions. Variants located near *SIX3* that are associated with levels of glucose are also associated with levels of expression of the genes *SIX3* and *SIX2* in pancreatic islets. These variants are located in a genomic region predicted to enhance gene expression. We used laboratory assays to show that alleles at one variant, rs12712928, demonstrate significant differences in transcriptional activity, suggesting that this variant influences levels of the *SIX3* and *SIX2* genes in islets, ultimately increasing glucose levels.

## Introduction

Type 2 diabetes (T2D) is a chronic disease affecting over 422 million people worldwide[1] with over 30% of cases occurring in East Asian populations [2]. Large-scale genome-wide association studies (GWAS) have identified >100 loci associated with T2D and >80 loci associated with fasting glucose, fasting insulin, and glycated hemoglobin (HbA1c), many of which have also been implicated in T2D susceptibility [3–6]. While the largest GWAS of glycemic traits and T2D to date have been performed in populations of predominantly European ancestry [3, 6–9], other studies have identified glycemic trait and T2D associations in East Asian individuals [5, 10, 11]. As glycemic trait profiles, allele frequencies, and environmental contributions differ between populations, continued investigation of genetic factors can discover additional loci influencing inter-individual variation in fasting glucose, fasting insulin, and HbA1c levels and T2D.

A new resource for genetic analyses, the China Health and Nutrition Survey (CHNS) is an ongoing, household-based, longitudinal survey aimed at examining economic, sociological, demographic, and health questions in a diverse Chinese population [12]. Using a multistage random-cluster design and stratified probability sampling to select counties and cities, data were collected from 228 communities across nine provinces (Guangxi, Guizhou, Heilongjiang, Henan, Hubei, Hunan, Jiangsu, Liaoning, and Shandong) that constituted 44% of China’s population as of the 2009 census. In addition to nearly 30 years of longitudinal survey data collected during 9 survey rounds from 1989–2011, quantitative biomarker measurements and DNA are available on 8,403 subjects in the CHNS.

Individual GWAS loci can harbor multiple association signals. More than one association signal has been reported at *G6PC2* and *PCSK1* for fasting glucose and at *KCNQ1*, *ANKRD55*, *CDKN2A/B*, *DGKB*, *HNF4A*, and *CCND2* for T2D [5]. Imputation reference panels generated from large sample sizes can facilitate identification of additional signals. For non-European populations, the 1000 Genomes Phase 3 reference panel is currently the most comprehensive, containing information for more than 88 million variants in >2,500 individuals from 26 diverse populations [13]. Identification of additional association signals at trait-associated loci could explain additional heritability and provide further insights into the biology between the locus and the trait or disease.

GWAS have been an efficient method for studying genetic factors influencing biological mechanisms underlying glycemic traits and T2D, but for many of the identified loci, the underlying gene(s), direction of effect, and disease mechanism are largely unknown [14]. For variants located in non-coding regions of the genome, bioinformatic datasets can be used to annotate and predict regulatory variants, target genes, and direction of effect [15–18], and these variants can be tested for allelic differences in regulatory activity with *in vitro* laboratory assays [19, 20]. For example, among previous functional studies of variants associated with fasting glucose at the *G6PC2-ABCB11* locus, two variants in the promoter were shown to affect *G6PC2* expression levels by altering FOXA2 binding, and two variants located in the third intron of *G6PC2* were shown to affect *G6PC2* splicing [21–23]. However, the majority of the glycemic trait-associated variants have not been examined.

To further clarify the genetic contributions to normal variation in glycemic traits in a multi-provincial Chinese population, we performed a GWAS of fasting glucose, insulin, and HbA1c levels and T2D in subjects from the CHNS, using genetic data imputed to 1000 Genomes Phase 3 in up to 7,178 subjects [12]. We examined the population substructure within the CHNS and evaluated candidate functional regulatory variants at one locus using annotation and *in vitro* laboratory assays.

## Results

### CHNS population structure

**Fig 1:**
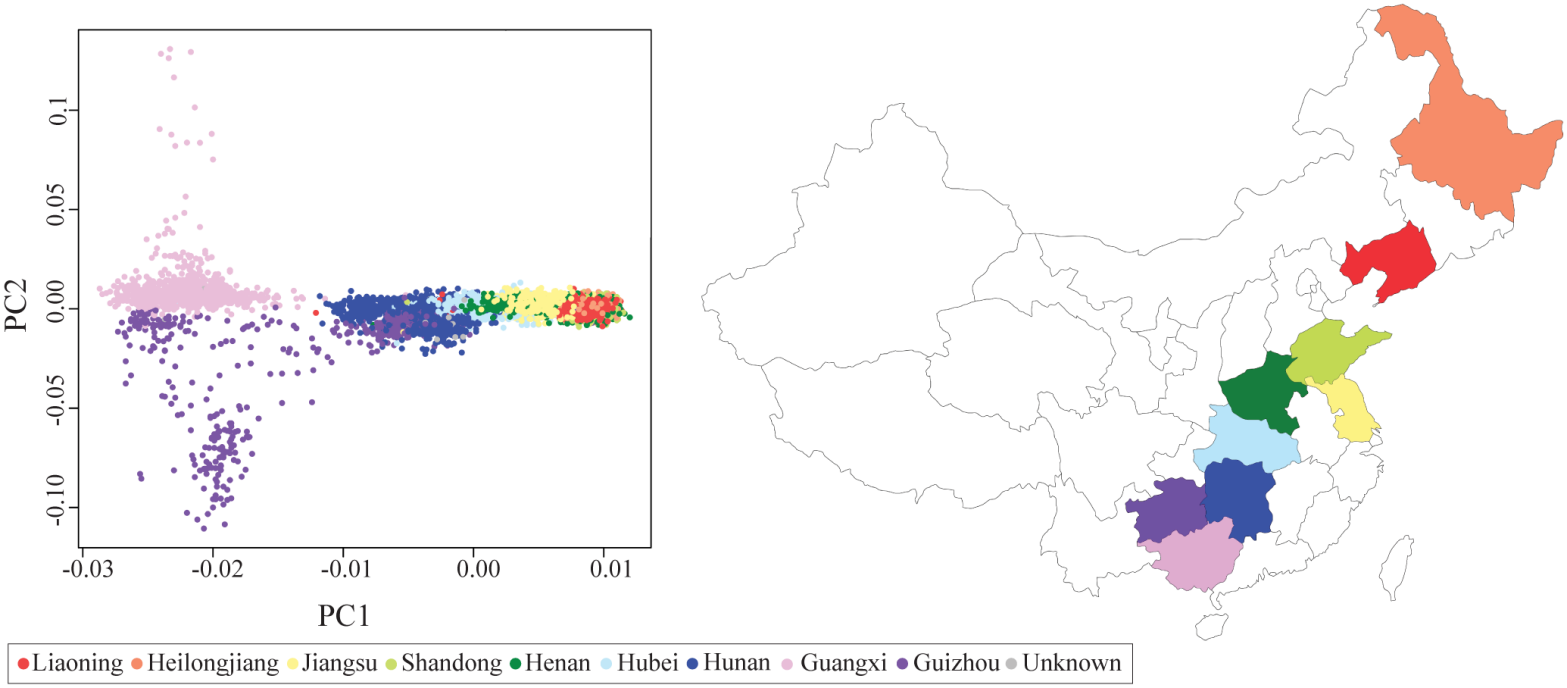
Principal components analysis of allele frequency for 8,403 subjects in the China Health and Nutrition Survey. Dots representing each subject are colored by the province in which they reside.

To evaluate population substructure among 8,403 CHNS subjects with genotype data available, we constructed principal components (PCs) using a subset of variants (MAF > 0.05; pairwise LD r^2^ <0.02 in a sliding window of 50 variants). Compared to HapMap 3 populations, the CHNS participants clustered closely with the Han Chinese in Beijing (CHB), the Han Chinese in Denver (CHD), and the Japanese in Tokyo (JPT) populations, with greater diversity than any of these populations (S1A Fig). A comparison to only the East Asian samples showed more clearly that the distribution of the CHNS extends beyond that of the CHB, CHD, and JPT samples (S1B Fig). Within the CHNS, the subjects showed two axes of diversity (Fig 1, S2 Fig). PC1, which explained 4.2% of the variance, appeared to cluster by province, while PC2, explaining 0.6% of the variance, showed diversity among subjects within the Guangxi and Guizhou provinces in southern China. PC2 could be partially explained by differences in self-reported ethnicity, particularly among subjects from the Guizhou province, as PC2 appeared to characterize the Miao and Buyi ethnic groups (S3 Fig). To account for population substructure in subsequent association analyses with glycemic traits, we included PC1 as a covariate and performed analyses using an efficient mixed model approach that accounts for sample structure between individuals [24].

### GWAS results

We performed genome-wide association analyses of fasting glucose and fasting insulin levels in up to 8,045,193 genotyped and imputed variants (MAF >0.01) from 5,786 non-diabetic individuals in the CHNS who provided fasting blood samples (S1 Table, S4 Fig). We also performed a genome-wide association analysis of HbA1c in 7,178 nondiabetic individuals who provided fasting or non-fasting samples. In addition, 5,731 unrelated subjects were used to assess variant association with T2D status, including 748 cases and 4,983 controls. For each trait, we also searched for additional signals by conditioning on the lead variants (reciprocal conditional analyses). Overall, a majority of CHNS subjects were female (54%) with a normal BMI (mean = 23.2 kg/m^2^), and subjects with T2D were older (cases: 59.7 years; controls: 51.2 years) with a higher BMI, higher fasting glucose levels, and higher fasting insulin levels (S1 Table).

**Fig 2:**
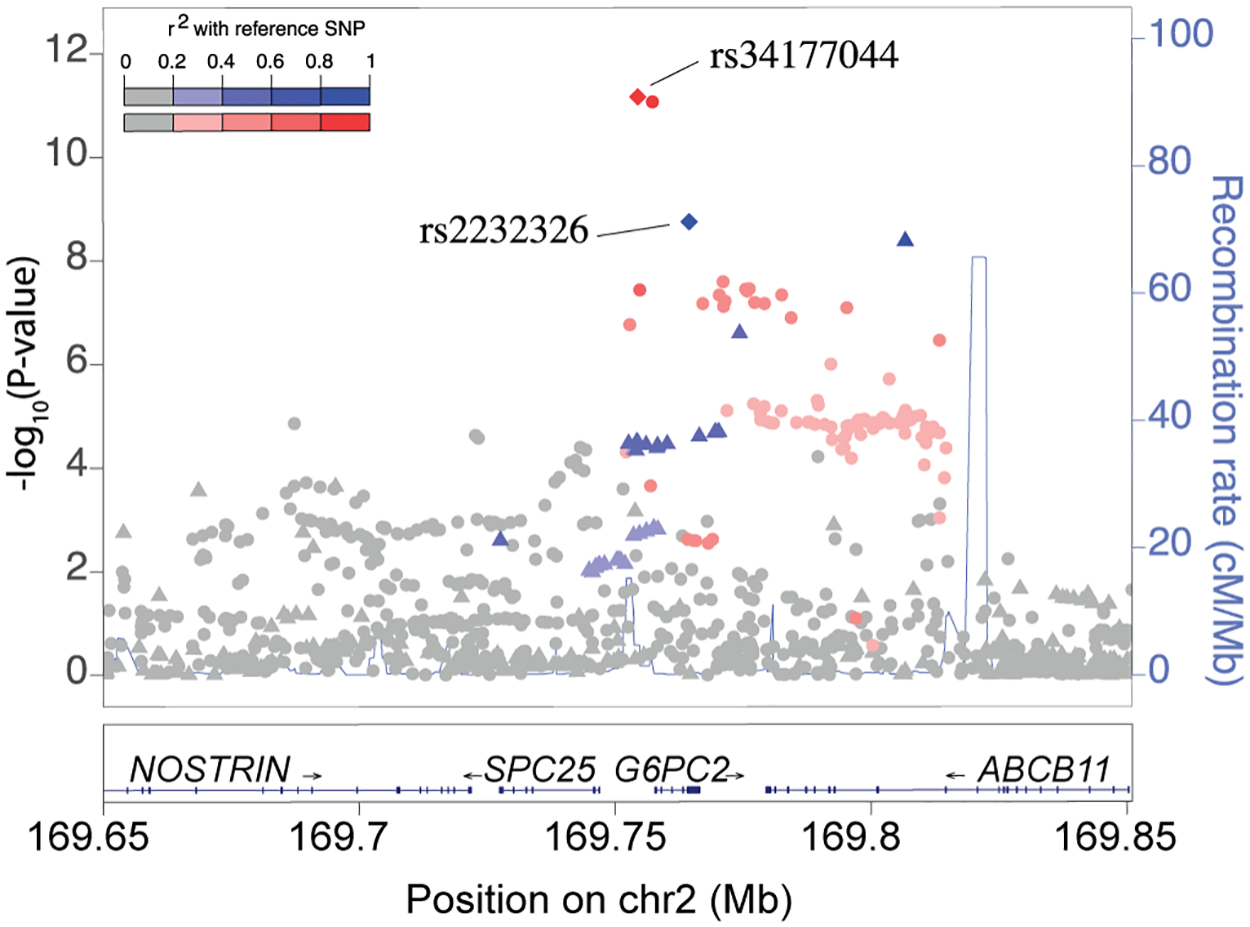
Fasting glucose locus near *G6PC2* exhibits two association signals in the CHNS. The first association signal, rs34177044 (red diamond) shows the strongest association in the initial unconditioned analysis of fasting glucose. Coding variant rs2232326 (S324P; blue diamond), remained locus-wide significant after conditioning on rs34177044. The diamonds indicate the lead variants, which exhibited the strongest evidence of association at the locus among 1000 Genomes Project Phase 3-imputed variants. Variants are colored based on LD with the lead variants, rs34177044 (red) and rs2232326 (blue) within 8,403 CHNS subjects.

**Fig 3:**
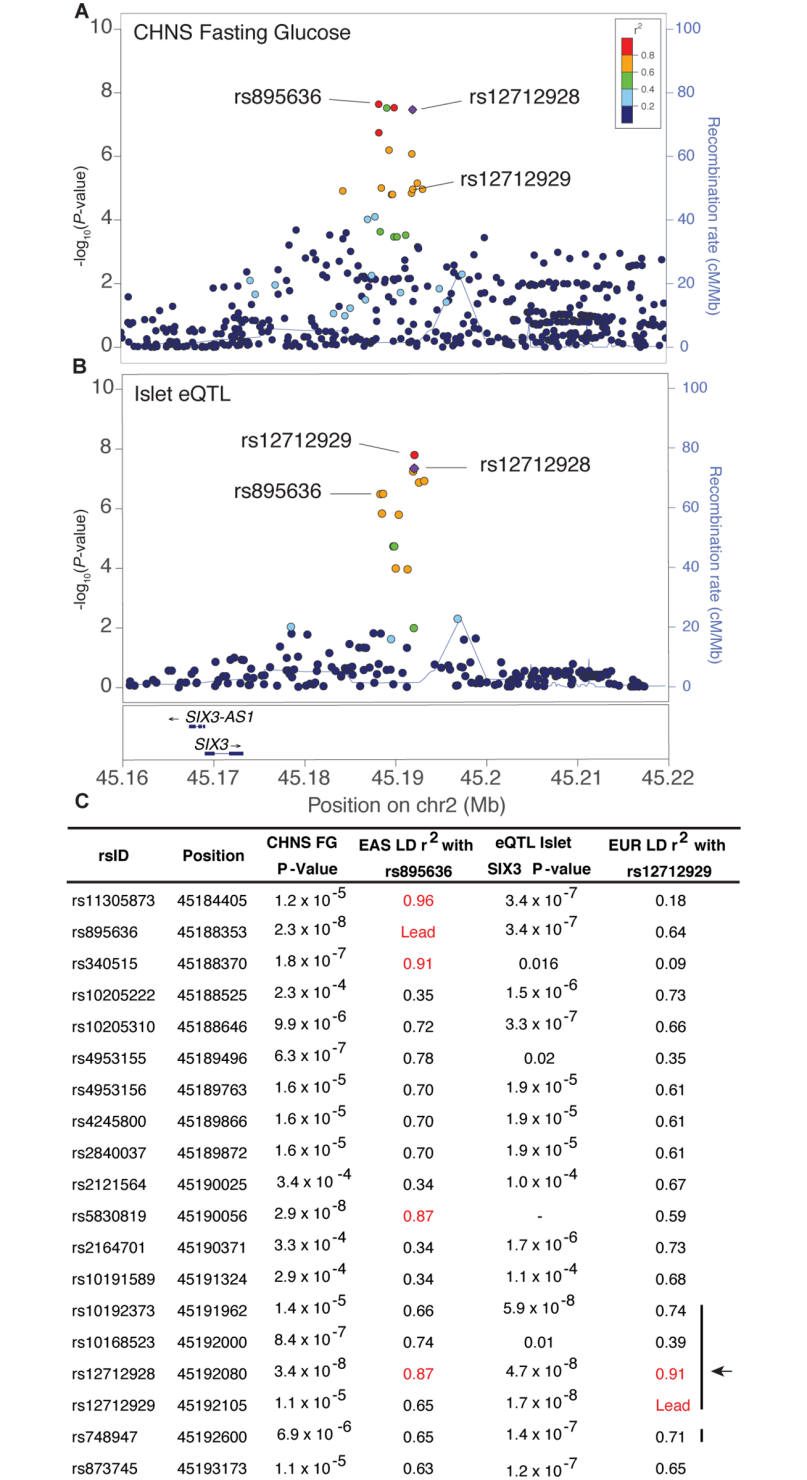
Pancreatic islet eQTL colocalizes with the fasting glucose GWAS locus. (A) rs895636 (purple diamond) shows the strongest association with fasting glucose in the CHNS. Variants are colored based on East Asian LD from 1000 Genomes Project Phase 3. (B) rs12712929 (purple diamond) shows the strongest association with expression of *SIX3* in pancreatic islets in European ancestry individuals. Variants are colored based on European LD from 1000 Genomes Project Phase 3. (C) Although the LD r^2^ between rs895636 and rs12712929 is moderate in both European and East Asian populations (1000G Phase 3), one variant, rs12712928, exhibits high LD (r^2^>0.80) with rs895636 in East Asians and rs12712929 in Europeans (arrow). Red font indicates variants above r^2^ of 0.80. Vertical bars indicate the genomic regions examined for allelic differences in regulatory function.

Analysis of fasting glucose confirmed eight loci previously identified in East Asian and European samples (*G6PC2*, *SIX3-SIX2*, *PROX1*, *ABCB11*, *GCK*, *KANK1*, *GLIS3*, and *TCF7L2*; S2 Table), two of which achieved genome-wide significance (rs34177044, near *G6PC2*, *P* = 6.9 x 10^−12^, Fig 2; rs895636, near *SIX3-SIX2*, *P* = 2.3 x 10^−8^, Table 1, Fig 3A, S5 Fig) [11, 25–27]. At these two loci, we used stepwise conditional analyses to identify additional association signals at a locus-wide threshold of *P* <1 x 10^−5^. Conditional analysis including rs34177044 at the *G6PC2* locus revealed a second signal (rs2232326, MAF = 0.04, *P*_*unconditioned*_ = 1.8 x 10^−9^, *P*_*conditioned*_ = 2.0 x 10^−6^, Fig 2). When conditioning only on rs2232326, rs34177044 was attenuated but remained significantly associated with fasting glucose (*P*_*conditioned*_ = 7.0 x 10^−9^); the attenuation suggests the two signals are distinct yet not fully independent. Haplotype analyses (S3 Table) and regression models containing an interaction term with both variants (*P* = 0.69) do not suggest a haplotype effect between the two signals, providing further evidence that the two signals are separate. While conditional analyses could be influenced by the moderate imputation quality of rs34177044 (r^2^ = 0.70) in CHNS, genotypes from the 1000 Genomes project show that the minor allele of rs2232326 is only inherited with the major allele of rs34177044 (East Asian LD r^2^ = 0.04, D’ = 1.0). No additional association signals were identified at the *SIX3-SIX2* locus (S5 Fig).

**Table 1:** Signals associated with levels of quantitative glycemic traits among non-diabetic subjects in CHNS.

Locus	SNP	Chr	Position	EA/NEA	EAF	Imputation r^2^	Genotype	Beta	SE	*P*-value
Fasting Glucose										
*SIX3-SIX2*	rs895636	2	45,188,353	T/C	0.40	GT	2124/2709/953	0.099	0.018	2.3 x 10^−8^
*G6PC2*	rs34177044	2	169,754,485	A/G	0.37	0.70	2043/2935/808	0.145	0.021	6.9 x 10^−12^
*G6PC2*	rs2232326	2	169,764,491	T/C	0.96	GT	5281/488/17	0.252	0.042	1.8 x 10^−9^
HbA1c										
*FN3KRP*	rs9895455	17	80,683,980	T/C	0.49	0.97	1821/3504/1853	0.095	0.018	1.0 x 10^−7^
Fasting Insulin										
*CNTN6*	rs13078376	3	932,902	C/T	0.69	0.69	2637/2688/484	0.124	0.024	3.2 x 10^−7^

Results are shown if the association signal reached genome-wide significance (*P* <5x10^-8^) or is the strongest association if no signals reached genome-wide significance. Trait values were adjusted for age, age^2^, BMI, PC1, and sex. Chromosome positions are based on hg19. Effect alleles are associated with higher trait values. Beta estimates reflect per allele effects of variants on inverse normal transformed traits. CHNS, China Health and Nutrition Survey; chr, chromosome; EA, effect allele; NEA, noneffect allele; EAF, effect allele frequency; SE, standard error; GT, genotyped

The lead variant in the second signal at *G6PC2* (rs2232326) is a missense variant (S324P). Amino acid 324 is located in a helix spanning the cell membrane [28], and the substitution of a proline for a serine in the middle of a helix may add kinks to the protein [29]. In addition, both SIFT and PolyPhen [30] predict this variant to be “probably damaging”, suggesting that it may affect function of the G6PC2 protein. Based on data from 1000 Genomes Phase 3, rs2232326 is rare in all ancestry populations (MAF: African, 0.2%; Admixed American, 0.3%; European, 0.3%; South Asian, 0.3%) except in East Asians (MAF 5%), and it has few (Admixed American, rs34102076; East Asian, rs139014876), to no (African, European, and South Asian) proxy variants (LD r^2^>0.80). This variant contributed to a significant *G6PC2* gene-based association with glucose in Europeans [31] and other protein-coding variants within *G6PC2* have been individually associated with fasting glucose levels (e.g. rs492594, rs138726309, rs2232323) [21]. In the CHNS, rs492594 was nominally associated with fasting glucose levels (*P* = 0.002); other previously described coding variants were either monomorphic or did not pass imputation quality control thresholds in CHNS (S4 Table).

We examined whether the strength of fasting glucose associations at *SIX3-SIX2* and *G6PC2* varied by province (Table 2). At rs895636 (*SIX3-SIX2)*, the minor allele frequencies (MAF) differed by as much as 0.12 between provinces. Most of the provinces in which individuals have a relatively lower minor allele frequency (0.35–0.38) showed a stronger association between the variant and fasting glucose levels than similarly sized samples of individuals with higher MAF of 0.43–0.47. The opposite pattern was observed at rs2232326 (*G6PC2*), for which the province in which the largest MAF (0.09) showed the strongest association with fasting glucose levels. The allele associated with higher levels of fasting glucose trended from less frequent in the northern provinces (MAF = 0.02) to more frequent in the southern provinces (MAF = 0.09). Although allele frequencies between provinces were not statistically different, observed allele frequency differences are consistent with genetic drift and the observed population substructure (Fig 1) [32], demonstrating that study samples from across China have differing power to detect specific associations.

**Table 2:** Comparison by province of genome-wide significant signals for fasting glucose.

Province	N	*SIX3-SIX2*			*G6PC2*					
		rs895636-T			rs34177044-A			rs2232326-C		
		MAF	Beta	*P*-value	MAF	Beta	*P*-value	MAF	Beta	*P*-value
Heilongjiang	736	0.35	0.134	0.007	0.35	0.194	0.0009	0.02	-0.199	0.22
Liaoning	564	0.37	0.036	0.48	0.36	0.172	0.006	0.02	-0.365	0.03
Shandong	625	0.36	0.152	0.003	0.40	0.156	0.01	0.03	-0.232	0.16
Jiangsu	723	0.38	0.125	0.004	0.39	0.086	0.11	0.04	-0.007	0.95
Henan	732	0.36	0.120	0.009	0.37	0.076	0.14	0.03	-0.405	0.0009
Hubei	349	0.43	0.086	0.25	0.38	0.130	0.14	0.04	-0.151	0.42
Hunan	660	0.44	0.057	0.21	0.36	0.084	0.13	0.05	-0.305	0.003
Guizhou	461	0.43	0.128	0.07	0.36	0.188	0.03	0.07	-0.178	0.22
Guangxi	936	0.47	0.057	0.14	0.36	0.157	0.001	0.09	-0.277	3.5 x 10^−5^
All Subjects	5,786	0.40	0.099	2.3 x 10^−8^	0.40	0.145	6.9 x 10^−12^	0.04	-0.252	1.8 x 10^−9^

Provinces are ordered based on geographical location from north to south. Trait values were adjusted for age, age2, BMI, PC1, and sex, and the residuals were then inverse normal transformed. Beta estimates reflect per allele effects of variants on inverse normal transformed traits. Only non-diabetic subjects were included in the analyses. Global tests for heterogeneity between the provinces were not statistically significant (*P*>0.05). CHNS, China Health and Nutrition Survey; MAF, minor allele frequency.

Analysis of fasting insulin validated (*P* <0.05) two loci previously reported in European and Hispanic samples (*PPARG* and *LOC284930*; S5 Table) [33, 34] and did not reveal any genome-wide significant loci (Table 1). The most significant association was identified near *CNTN6* (rs13078376, *P* = 3.22 x 10^−7^; Supplementary Materials, S6 Fig). Previous studies have demonstrated genome-wide significant associations between variants approximately 1 Mb upstream of the *CNTN6* gene and both fasting insulin (rs9841287) [33] and HbA1c levels (rs892295) [35], although the rs13078376 is not a proxy for either of the two previously reported variants (East Asian LD r^2^<0.01). Data from the CHNS strengthen the evidence for these nominally significant loci near *CNTN6*.

Analysis of HbA1c validated (*P* <0.05) nine loci previously identified in East Asians and Europeans (*FN3KRP*, *MYO9B*, *PIEZO1*, *ANK1*, *GCK*, *SPTA1*, *HBS1L*, *MTNR1B*, and *ABCB11*; S6 Table) [10, 36, 37], and did not reveal any genome-wide significant loci. The most significant variant was located within an intron of *FN3KRP* (rs9895455, *P* = 3.5 x 10^−7^; Supplementary Materials, S7 Fig). rs9895455 is in high LD (East Asian, r^2^ = 0.99) with a variant previously reported to be associated with HbA1c in East Asians (rs1046875) [10]. Three additional variants in high LD with rs9895455 have previously demonstrated moderate associations in Europeans with both HbA1c (*P* = 4.1x 10^−7^) and modified Stumvoll insulin sensitivity index (*P* = 0.02) [38]. While power to detect associations is limited, data from the CHNS provide further support for these established loci.

Association analyses for T2D validated (*P* <0.05) sixteen loci previously identified in East Asians and/or Europeans (*POU5F1/TCF19*, *SLC30A8*, *CUBN*, *MIR17HG*, *TMEM18*, *GLP2R*, *GIPR*, *MC4R*, *BCL2L11*, *PAX4*, *IGF2BP2*, *PRC1*, *KCNQ1*, *CDKN2A/B*, *TLE4*, and *PAM/PPIP5K2*; S7 Table) [4, 26, 39–41], and did not reveal any genome-wide significant loci. Of the validated loci, the most significant are at *SLC30A8* (rs3802177, *P* = 3.0 x 10^−4^) and *POU5F1/TCF19* (rs2073721, *P* = 2.3 x 10^−4^). The three most significant variants not described previously were located near *RTN4RL1* (rs62069176, *P* = 2.7 x 10^−7^), *SOCS6*, (rs2581685, *P* = 2.6 x 10^−6^), and *ARID1B* (rs6557473, *P* = 3.3 x 10^−6^) (S8 Table, S8 Fig). Of these, prior suggestive associations (*P*<0.05) have been detected previously between rs6557473 at *ARID1B* and both fasting insulin and T2D in Europeans [9, 42, 43]. The CHNS data provide suggestive evidence for these loci.

### Annotating association signals with eQTL data

To aid in the identification of candidate genes at the strongest association signals, we examined whether any of the variants associated with glycemic traits are also associated with expression of nearby transcripts in pancreatic islets, blood, subcutaneous adipose, or tissues from GTEx (S9 Table) [44–46]. These expression quantitative trait locus (eQTL) datasets were generated predominantly from European ancestry donors. GWAS and eQTL signals more clearly coincide when the GWAS variant and the variant most strongly associated with expression level of the corresponding transcript exhibit high pairwise LD (r^2^>0.80). To allow for differences in LD patterns across ancestries in the GWAS and eQTL datasets, we considered GWAS and eQTL signals to be possibly coincident at a less stringent threshold for pairwise LD values (r^2^>0.60, East Asian 1000G Phase 3). The association signal for fasting glucose in East Asians at the *SIX3-SIX2* locus contained fifteen variants meeting this criterion (lead GWAS variant rs895636 and fourteen variants with East Asian LD r^2^≥0.60), and the association signal for islet *SIX3* expression in Europeans contained 14 variants (lead variant and 13 variants with European LD r^2^≥0.60) (Fig 3B, S9 Table). One variant, rs12712928, exhibited high LD (r^2^>0.80) with both the lead GWAS and eQTL variants (Fig 3C). rs12712928-C showed strong association with higher fasting glucose (*P* = 3.4 x 10^−8^), similar to the lead fasting glucose GWAS variant (rs895636, *P* = 2.3 x 10^−8^), and strong association with lower *SIX3* expression level in pancreatic islets (*P* = 4.7 x 10^−8^), similar to the lead *SIX3* eQTL variant (rs12712929, *P* = 1.7 x 10^−8^). In addition to *SIX3*, rs12712928-C was strongly associated with lower expression level of *SIX2* (*P* = 1.4 x 10^−4^), *SIX3-AS1* (*P* = 4.8 x 10^−6^), and two other long non-coding transcripts (S9 Table) [47]. Assuming the fasting glucose GWAS and islet eQTL signals are shared across ancestries, then the strongest candidate variant that may be responsible for both associations is rs12712928.

### Regulatory function of prioritized candidate regulatory variants

To establish a set of candidate functional variants at the *SIX3-SIX2* locus, we used regulatory chromatin marks (open chromatin and histone states) to predict which variants may affect the transcription of nearby genes. Of 19 candidate variants at the *SIX3-SIX2* locus (including the lead GWAS variant rs895636, the lead islet eQTL variant rs12712929, variants in EAS LD r^2^>0.60 with rs895636, and variants in EUR LD r^2^>0.6 with rs12712929), only five variants (rs10192373, rs10168523, rs12712928, rs12712929, and rs748947) overlap pancreatic islet active enhancer and pancreatic islet open chromatin (DNase or FAIRE) marks, as well as predicted transcription factor binding motifs (S9 Fig). All five of these variants have EAS LD r^2^ 0.66–0.87 with lead GWAS variant rs895636. These data suggest that these five variants are the strongest candidates to affect transcription of the gene(s) at this locus.

**Fig 4:**
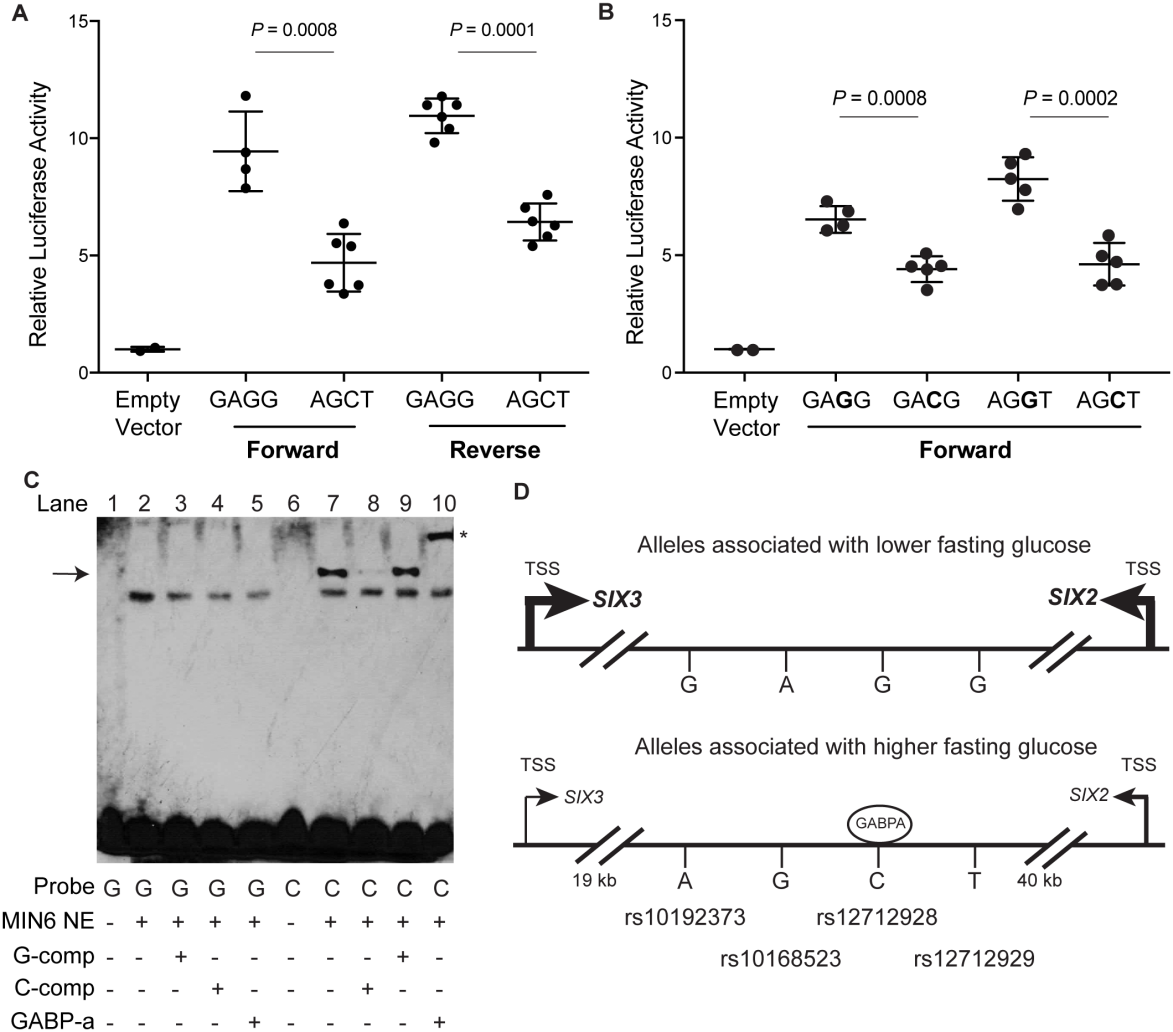
rs12712928 exhibits allelic differences in transcriptional activity and protein binding. (A) A haplotype of four variant alleles associated with lower fasting glucose, GAGG, at rs10192373, rs10168523, rs12712928, and rs12712929 repeatedly showed greater transcriptional activity in both forward and reverse orientations with respect to *SIX3* in MIN6 mouse insulinoma cells compared to the AGCT haplotype and an “Empty Vector” containing a minimal promoter. (B) Analysis of additional haplotypes created by site-directed mutagenesis of rs127129258 alleles show that haplotypes containing, rs12712928-C (GA**C**G and AG**C**T), exhibited less transcriptional activity than haplotypes containing rs12712928-G (GA**G**G and AG**G**T) in the forward orientation. (C) EMSA with biotin-labeled probes containing the C or G allele of rs12712928 show an allele specific band (arrow; lane 7 versus 2) that is competed away more effectively by 45-fold excess of unlabeled probe containing the C allele (lane 8) than the G allele (lane 9). An arrow points to an allele-specific protein complex binding to the C allele. We observed a supershift with the addition of antibodies to the alpha subunit of GABP (denoted by *). (D) Model of rs12712928 as a functional regulatory variant at the *SIX3-SIX2* locus. Alleles, including rs12712928-C, are associated with higher fasting plasma glucose levels and lower expression of *SIX3* and other transcripts in human pancreatic islets. Arrows indicate the transcription start site (TSS) of the *SIX3* and *SIX2* genes. An oval represents GABP bound differentially to rs12712928-C, which exhibited lower transcriptional reporter activity compared to rs12712928-G.

To evaluate the allelic differences in enhancer activity of the five candidate functional variants, we conducted transcriptional reporter assays in MIN6 mouse insulinoma cells. We tested 4–6 independent constructs corresponding to each allele or haplotype for a 312-bp DNA region located 18 kb downstream of *SIX3* and 37 kb from the 3’ end of *SIX2* spanning rs10192373, rs10168523, rs12712928, rs12712929 (tested as a haplotype) and for a 365-bp region located 20 kb from the 3’ end of *SIX3* spanning rs748947 (S9 Fig). While the rs748947 construct showed no enhancer or allele-specific activity (S10 Fig), the haplotype construct had haplotype-differences in enhancer activity in both orientations (Fig 4A). This 4-variant construct containing the fasting glucose-increasing alleles (rs10192373-A, rs10168523-G, rs12712928-C, and rs12712929-T) showed significantly decreased enhancer activity of ≥ 1.4-fold in magnitude (forward, *P* = 0.0008; reverse, *P* = 0.0001) compared to the haplotype containing the non-risk alleles. To determine whether rs12712928 could account for the allele-specific effects, we used site-directed mutagenesis to create two additional haplotype constructs. Haplotype constructs containing rs12712928-C exhibited a 1.5-fold decrease in enhancer activity compared to the haplotype constructs containing rs12712928-G (Fig 4B). Taken together, these data show that rs12712928 exhibits allelic differences in transcriptional enhancer activity and suggest it functions within a *cis*-regulatory element at the *SIX3-SIX2* fasting glucose-associated locus.

We next asked whether alleles of rs12712928 or the other three variants differentially affect DNA binding to nuclear proteins. A DNA-protein complex specific to the rs1272928-C allele was observed using electrophoretic mobility shift assays (EMSA) with MIN6 nuclear lysate (S10 Table, S11–S12 Figs). Competition with excess unlabeled C-allele probe more efficiently competed away allele-specific bands than excess unlabeled G-allele probe, providing further support for allele-specificity of the protein-DNA complexes (Fig 4C). Based on these results, we hypothesized that rs12712928-C is located in a binding site for a transcriptional regulatory complex that may be disrupted by the rs12712928-G allele. The sequence containing rs12712928-C is predicted to include a consensus core-binding motif for several transcription factors, and a ChIP-seq peak for CTCF also overlaps this region [48–50]. To identify transcription factor(s) binding to rs12712928, we used a DNA-affinity capture assay. A protein band showing allele-specific binding to the C allele was identified as the alpha subunit of GABP using MALDI TOF/TOF mass spectrometry. In EMSA supershift assays using antibodies to GABP-α, we observed a supershift of the allele-specific band (Fig 4C), suggesting that GAPB may act as a repressor to reduce enhancer activity at this locus (Fig 4D).

We used a similar approach to identify potentially functional variants at the *G6PC2* locus. The first signal is comprised of two intergenic variants (GWAS index variant and one variant in LD r^2^ >0.80). GWAS variant, rs34177044, is ~3.2 kb upstream from the transcription start site of *G6PC2* and does not overlap any predicted open chromatin marks. rs1402837 (LD r^2^ = 0.97) is located 646 bp upstream of the *G6PC2* transcription start site and 187 bp and 208 bp upstream, respectively, of other promoter variants previously shown to exhibit allelic differences on transcriptional activity, rs573225 and rs2232316 (S13 Fig) [51]. rs1402837 also overlaps open chromatin marks, suggesting rs1402837 may play a regulatory role in fasting glucose levels in the context of other *G6PC2* promoter variants.

## Discussion

In this study of genetic associations with T2D and related glycemic traits in Chinese individuals from 9 provinces in the CHNS, we observed associations with fasting glucose at *SIX3-SIX2* and *G6PC2*, including a coding variant representing an additional signal at *G6PC2*. We also showed that the *SIX3-SIX2* fasting glucose locus colocalizes with eQTL associations for *SIX3*, *SIX2*, *SIX3-AS1*, *RP11-89K21*.*1*, and *AC012354*.*6* in pancreatic islets, and we showed evidence that rs12712828 functions as a regulatory variant at the *SIX3-SIX2* fasting glucose locus. Genetic associations in CHNS also supported (*P*<0.05) previously reported associations at 6, 2, 9, and 16 loci with fasting glucose, fasting insulin, HbA1c, and T2D, respectively. The moderate sample size of CHNS prohibited us from identifying additional associations.

Hundreds of genes contribute to the heritability of complex traits [52]. As more GWAS and genome-wide meta-analyses are conducted across genetically diverse populations, identification of additional association signals and loci will help to explain the levels of heritability. The CHNS adds to the growing number of population-based cohorts available for the study of metabolic traits. With its multi-provincial study design, the CHNS includes subjects of differing ethnicities, from both urban and rural areas across China. Additionally, linkage of the genotype data with biomarkers and decades of longitudinal phenotype data (e.g. nutrition, health outcomes, environment) will allow environmental and societal contributions to trait or disease outcomes to be evaluated.

Alterations in regulatory elements or the coding sequence of *G6PC2* can impact levels of fasting plasma glucose. *G6PC2* encodes an enzyme belonging to the glucose-6-phosphatase catalytic subunit family responsible for the terminal step in gluconeogenic and glyconeogenic pathways that lead to the release of glucose into the bloodstream [53]. Several previous studies have identified >1 fasting glucose association signals at this locus in populations of European and African ancestries, two of which include nonsynonymous coding variants [25, 26, 31, 33, 54–57]. We identified two distinct signals at *G6PC2* associated with fasting plasma glucose levels. Variants within the primary CHNS association signal have been associated with fasting glucose in East Asian populations previously [11]. The lead variant in the second signal at *G6PC2* (rs2232326) is a missense variant (S324P). We were unable to assess evidence of association with other coding variants in *G6PC2* as the variants were either monomorphic in CHNS or did not pass quality control thresholds.

To date, the association between variants near *SIX3* and glycemic traits remains specific to East Asian populations. rs895636 was reported as the lead variant associated with fasting plasma glucose in a GWAS of >17,000 Korean and Japanese subjects (*P* = 9.9x10^-13^) and in a separate GWAS meta-analysis of up to 46,085 East Asians (*P* = 2.5x10^-13^) [27]. However, in Europeans, nominal to no association has been observed between rs895636 and fasting glucose (*P* = 0.002, n>96,000) [33], HbA1c (*P* = 0.05, n>46,000) [36], fasting insulin (*P* = 0.73, n>96,000) [33], and T2D (*P* = 0.41, n>120,000) [3]. Allele frequency is a possible explanation for ancestry differences. In East Asians, the MAF of rs895636 is 0.42 while the MAF in Europeans is only 0.16. Larger sample sizes of European-ancestry individuals may be needed to identify the association between variants at the *SIX3-SIX2* locus and glycemic traits. Other genetic and environmental factors may also be playing a role in the fasting glucose association at *SIX3-SIX2* in East Asian populations that are not present in other populations.

We provide compelling evidence that rs12712928 is a regulatory variant at the *SIX3-SIX2* fasting glucose locus. The rs895636-T allele is associated with increased fasting glucose levels and decreased *SIX3*, *SIX2*, and other transcript expression in pancreatic islets [47]. rs12712928 is in high LD (East Asian and European r^2^>0.87) with both rs895636 and the lead pancreatic islet eQTL variant, rs12712929, and was the strongest candidate for an effect of regulatory function based on its location in a putative islet enhancer element. Compared to rs12712928-G, the allele rs12712928-C demonstrated decreased transcriptional activity, as well as allele-specific binding to the alpha subunit of GABP, which suggests that at least GABP, and possibly other transcription factors, bind to the C allele and repress expression of *SIX3* and *SIX2*. rs12712928 may be responsible for the GWAS signal, or given that some GWAS signals are affected by multiple functional variants [58, 59], other variants at this locus may also contribute to variation in fasting glucose.

*SIX3* is a strong candidate for a target gene at the *SIX3-SIX2* fasting glucose locus. Highly expressed in pancreatic islets [44], *SIX3* encodes sine oculis homeobox-like protein 3, a transcription factor that localizes to the nucleus of adult beta cells to regulate insulin production and secretion. Decreased expression of *SIX3* results in the misregulation (i.e. decreased levels) of insulin [60], which promotes the uptake of glucose into fat, liver, and skeletal muscle cells, thus lowering blood glucose levels [61]. Consistent with the effects of *SIX3* in mice, the risk allele rs12712928-C is associated with both decreased expression of *SIX3* and increased levels of fasting glucose. In CHNS, rs12712928-C was also moderately associated with decreased fasting insulin levels (*P* = 7.6x10^-5^) and an increased risk for T2D (*P* = 0.03). However, in the islet eQTL data, rs12712928 was also associated with expression level of *SIX2*, *SIX3-AS1*, *RP11-89K21*.*1*, and *AC012354*.*6*. *SIX2* is also believed to play a role in the regulation of islet beta cell functions such as insulin output [60]; however, less is known about its biologic function compared to *SIX3*. Additionally, the roles of *SIX3-AS1*, *RP11-89K21*.*1*, and *AC012354*.*6* are not well characterized. One or more of these transcripts could be a target gene underlying the association signal and contributing to the biological effect on fasting glucose.

In conclusion, this study confirmed many previously identified loci associated with T2D and related glycemic traits and validated a recently described *G6PC2* missense variant associated with fasting glucose. We report a functional variant at the *SIX3-SIX2* locus, rs12712928, and provide evidence of a potential mechanism by which this variant affects expression of at least *SIX3*, leading to decreased levels of fasting glucose. Our use of a denser reference panel of >8 million variants in a diverse Chinese population allowed us to conduct higher resolution genetic analyses than reported previously. Further functional analyses of the variants identified in this study is the next step to confirm which variants and genes are affected. Replication of the moderately-significant associations would be useful to better understand the genetic architecture of glycemic traits.

## Materials and methods

### The China Health and Nutrition Survey

The China Health and Nutrition Survey (CHNS) is a nationwide, longitudinal survey aimed at examining economic, sociological, demographic, and health questions in a Chinese population. Details of subject selection and study design have been described elsewhere [12]. Briefly, a stratified probability sample with a multistage, random cluster design was used to select counties and cities within 9 diverse provinces (Guangxi, Guizhou, Heilongjiang, Henan, Hubei, Hunan, Jiangsu, Liaoning, and Shandong), stratified by income and urbanicity using State Statistical Office definitions. A total of 4,560 households from 228 communities were then randomly selected from within each stratum. Health data was collected during nine rounds of surveys from 1989–2011 (1989, 1991, 1993, 1997, 2000, 2004, 2006, 2009, and 2011). The 2009 survey was the first to collect fasting blood samples. The CHNS was approved by the Institutional Review Boards at the University of North Carolina at Chapel Hill (#07–1963, #05–2369), the Chinese National Human Genome Center at Shanghai (#2017–01), and the Institute of Nutrition and Food Safety at the China Centers for Disease Control (#201524–1). All participants provided written informed consent.

The present analysis was limited to subjects who participated in the 2009 survey round and for whom blood biomarker traits were available (n = 9,551). For the glucose and insulin analyses, subjects were only included if their blood sample was obtained after an overnight fast (n = 6,779). Subjects were excluded from a particular analysis if their biomarker trait value exceeded 4 standard deviations beyond the group mean or have type 1 diabetes. Fasting blood samples were not required for the HbA1c or T2D analysis. Additionally, one member of each first-degree relative pair was randomly removed for analyses of T2D, as current software to analyze associations with binary traits do not control for the high number of related individuals with CHNS.

### Glycemic trait measurements

Following an overnight fast, a blood sample (12 mL) was collected by venipuncture. Glucose and insulin were measured in the central laboratory of the China-Japan Friendship Hospital. Detailed descriptions of the laboratory procedures for measuring glucose (GOD-PAP method; Randox Laboratories Ltd, UK), and insulin (radioimmunology in a gamma counter, XH-6020 analyzer; North Institute of Bio-Tech, China), levels have been described previously [62]. HbA1c was measured in the central laboratory of the China-Japan Friendship Hospital [Guizhou and Hunan, HLC method, HLC-723 G7 (machine), Tosoh, Japan (reagent). Theory: Boronic Acid Afinity HPLC] or in the field [Guangxi and Henan: HPLC method, Primus Ultra 2 (PDQA1c), Primus, USA; Heilongjiang, Hubei, Jiangsu, and Liaoning: HLP method, Bio-Rad (D10), Bio-Rad, USA; Shandong: HLC method, HLC-723 G7, Tosoh, Japan. Theory: Ion exchange HPLC]. Different methods and machines were calibrated with the same quality control products made in Bio-Rad, USA. Participants were classified as having T2D if they were at least 18 years old and met at least one of the following criteria: 1) HbA1c ≥6.5%, 2) fasting blood glucose ≥126 mg/dL, 3) received the diagnosis from a physician after the age of 20 (self-report), or 4) reported taking diabetes medication (self-report) (S1B Table).

### Genotyping and quality control

DNA samples were extracted and genotyped at the Chinese National Human Genome Center, Shanghai, China. Genotyping was performed with the Illumina HumanCoreExome chip using the standard protocol recommended by the manufacturer. Genotyping was attempted on the 10,131 unique samples, 316 duplicates, and 1 set of triplicates (total n = 10,449). 1,513 samples were unable to be genotyped due to inadequate DNA concentrations (<10 ng/uL or OD 260/280 outside the 1.5–2.0 range), and an additional 69 samples were excluded for poor quality. Using KING, we identified 7 pairs of samples that were unintentionally duplicated; one sample from each pair was excluded. We used PLINK v.1.9 to compare genotype heterozygosity on the X chromosome to self-reported gender and excluded 129 mismatched samples and six samples with apparent XXY or XXXY genotypes. The CHNS data contained 4 sets of identical twins; 1 subject was randomly excluded from each twin pair. Finally, based on the principal component analysis described below, we excluded two samples that were outliers from HapMap samples of Han Chinese in Beijing, China (CHB), Chinese in Metropolitan Denver, Colorado (CHD), and Japanese in Tokyo, Japan (JPT). After exclusions, 8,403 samples were successfully genotyped and passed all genotyping quality control.

We applied variant quality control checks in PLINK v.1.9 on the 8,403 remaining CHNS samples that were successfully genotyped. Of the initial 538,448 variants, we discarded 4,306 variants due to call rate <95% and/or deviation from Hardy-Weinberg equilibrium (*P* <10^−6^). Of the remaining 534,143 variants, 193,236 (36.2%) were monomorphic and 340,906 (63.8%) were polymorphic.

Because of the household-based study design of CHNS, we expected the CHNS to include many first- and second-degree relatives. Using KING [63], we calculated kinship coefficients for all pairwise relationships and identified 3,681 first-degree relative pairs and 1,567 second-degree relative pairs.

### Genotype imputation

We performed genotype imputation of the autosomal chromosomes of 8,403 samples with the 1000 Genomes Project Phase 3 v5 reference panel [13, 64] using the Michigan Imputation Server [65]. We used Eagle2 [66] for pre-phasing, followed by imputation with Minimac3 software. We also imputed the X chromosome using with the 1000 Genomes Project Phase 3 v5 reference panel. We imputed male (n = 3,927) and female (n = 4,476) samples separately, using Mach for pre-phasing and Minimac2 for imputation. Imputation yielded data for 47,095,001 variants, and the 534,143 directly genotyped variants were also assigned imputed genotypes. We removed variants with an imputation r^2^ <0.30 (35,615,501 variants) or a MAF <0.01 (37,891,969 variants) as additional quality control procedures. In total, we tested 8,045,193 variants for association with fasting glucose, insulin, and HbA1c levels and T2D.

### Accounting for population substructure

We constructed principal components (PCs) to capture population substructure among the CHNS subjects. We identified a set of 55,601 independent variants with MAF > 0.05 and pairwise LD r^2^ <0.02 in a sliding window of 50 variants and used the variants to construct PCs in 8,403 CHNS subjects (Fig 1; S1–S3 Figs). The set of 55,601 variants was trimmed to match a list of 47,032 variants that were also available in HapMap Phase III samples. Individuals from CHNS and HapMap III were plotted based on the first two eigenvectors produced by the PC analysis (S1 Fig). We tested for the association between each of the first 10 PCs and each of the phenotypic traits to identify PCs associated at *P*<0.05; the first PC was included as a covariate in the regression models.

### Genome-wide association analysis

Fasting glucose, fasting insulin, HbA1c, and T2D were adjusted for age, age^2^, BMI, gender, and PC1. Residuals were then inverse normal-transformed to satisfy model assumptions of normality. Efficient mixed model associations (EMMAX) accounting for population structure and relatedness were performed using EPACTs v.3.1.0 [24]. Because EMMAX was designed for analysis of linear traits, GWA analyses for T2D were performed using the Firth bias-corrected logistic regression likelihood ratio test implemented in EPACTs [67]. For all analyses, genotype was modeled as an additive effect, with the genotype dosage values used as the primary predictor of interest. Due to the correlation of the glycemic traits, we used a genome-wide significance threshold of *P* <5 x 10^−8^ to define a single result as genome-wide significant, as used in previous association studies of this scale and high trait correlation [68]. A conservative experiment-wide Bonferroni-corrected *P*-value for four could be considered as *P*<1.25x10^-8^. We created regional association plots using LocusZoom [69] with LD estimates generated from the CHNS subjects. All variant positions correspond to build hg19.

### Conditional analyses

At loci that exhibited evidence of genome-wide significant association (*P* <5 x 10^−8^), we identified additional association signals using conditional analysis. We added the most strongly associated variant into the regression model as a covariate and tested all remaining regional variants (+/- 1 Mb from the initial lead GWA variant at each locus) for association. Since we were focusing on a much narrower region of variants during the conditional analyses, we set a less stringent locus-significance threshold of *P* <1 x 10^−5^ based on ~5,000 variants in a 2 Mb region. We performed sequential conditional analyses until the strongest variant no longer met the *P*-value threshold.

### Associations with other metabolic traits and outcomes

We used summary data available in the Type 2 Diabetes Knowledge Portal [70] to explore associations between the newly identified loci and other metabolic traits and outcomes. Association summary statistics available (last assessed June 23, 2017) included coronary artery disease from CARDIoGRAM [71]; kidney-related traits from CKDGen [72]; T2D from DIAGRAM, GoT2D, BioMe AMP, CAMP, and SIGMA [3, 43, 73–75]; BMI and waist-hip-ratio from GIANT [76, 77]; and glycemic traits from MAGIC [26, 36, 78, 79]. Additionally, we used data available from the ICP-GWAS (systolic and diastolic blood pressure) [80] and the AGEN adiponectin GWAS [81]. To identify variants in high LD (r^2^>0.80) with the lead variants, we used LDlink with all East Asian sample populations from the 1000 Genomes Project as the reference [82].

### Regulatory element annotation

We used ENCODE [15], ChromHMM [83], and Human Epigenome Atlas [84] data available through the UCSC Genome Browser to determine which of the candidate variants in each association signal overlapped open-chromatin peaks, ChromHMM [83] chromatin states, and chromatin-immunoprecipitation sequencing (ChIP-seq) peaks of histone modifications H3K4me1, H3K4me3, and H3K27ac, and transcription factors in pancreatic islets and the pancreas.

### Expression quantitative trait loci (eQTL)

We searched the following publicly available eQTL databases to identify *cis*-eQTLs at the observed loci: GTEx v7 [85], the University of Chicago eQTL browser [86], the Islet eQTL Explorer (http://theparkerlab.org/tools/isleteqtl/) [47], and the Blood eQTL Browser [45]. We also searched for *cis*-eQTLs in subcutaneous adipose tissue from the METSIM study [87]. All eQTL data sources used a false discovery rate (FDR) <5% for identifying *cis*-eQTLs, with the exception of the METSIM study, which used an FDR <1%.

### Cell culture

MIN6 mouse insulinoma cells[88] were cultured in DMEM (Sigma) supplemented with 10% FBS, 1 mM sodium pyruvate, and 0.1 mM beta-mercaptoethanol. The cell cultures were maintained at 37°C with 5% CO_2_.

### Transcriptional reporter assays

To measure variant allelic differences in enhancer activity at the *SIX3-SIX2* locus, we designed oligonucleotide primers (S10 Table) with KpnI and XhoI restriction sites, and amplified the 312-bp DNA region (GRCh37/hg19 –chr2: 45,191,902–45,192,213) around: rs10192373, rs10168523, rs12712928, rs12712929 (tested as a haplotype). Separately, we amplified a 365-bp region (GRCh37/hg19 –chr2:45,192,357–45,192,721) around rs748947. The 312-bp haplotype construct was altered to create a missing haplotype for rs12712928 using the QuickChange site directed mutagenesis kit (Stratagene). As previously described [19], we ligated amplified DNA from individuals homozygous for each allele into the multiple cloning site of the luciferase reporter vector pGL4.23 (Promega) in both orientations with respect to the genome. Isolated clones were sequenced for genotype and fidelity. 2x10^5^ MIN6 cells were seeded per well, and grown to 90% confluence in 24-well plates. We co-transfected five independent luciferase constructs and *Renilla* control reporter vector (phRL-TK, Promega) using Lipofectamine 2000 (Life Technologies) and incubated for another 48 hours. 48 hours post-transfection, the cells were lysed with Passive Lysis Buffer (Promega). Luciferase activity was measured using the Dual-luciferase Reporter Assay System (Promega) per manufacturer instructions and as previously described [19].

### Electrophoretic mobility shift assay (EMSA)

Nuclear cell protein was extracted from MIN6 cells using the NE-PER nuclear extraction kit (Thermo Scientific). 17 bp oligonucleotide probes were designed centered on each variant: rs10192373, rs10168523, rs12712928, and rs12712929 (S10 Table). The annealed double-stranded oligonucleotide biotin labeled and unlabeled probes for both alleles were generated as previously described [19]. To conduct EMSAs, we used the LightShift Chemiluminescent EMSA Kit (ThermoFisher Scientific) and followed the manufacturer’s recommendations. Briefly, a 20 μl binding reaction consisting of 6 μg nuclear extract, 1X binding buffer, 50 ng/μL poly (dI-dC), and 200 fmol of labeled probe was incubated at room temperature for 25 minutes. For competition reactions, 25-fold excess of unlabeled probe for either allele were incubated for 15 min prior to the addition of 200 fmol labeled probe and incubated for an additional 25 minutes. For supershift assays, 6 μg of polyclonal GABP-α antibody (sc28312X; Santa Cruz Biotechnology) was added to the binding reactions and incubated for 25 minutes prior to the addition of 200 fmol labeled probe. The reaction was further incubated for an additional 25 minutes. Protein-probe complexes were resolved on non-denaturing PAGE on 6% DNA retardation gels (Thermo Scientific), transferred to Biodyne B nylon membranes (PALL Life Sciences), cross-linked on a UV-light cross linker (Stratagene), and detected by chemiluminescence. EMSAs were carried out on second independent day and yielded comparable results.

### Identification of proteins binding rs12712928

To identify factors in the protein complex binding rs12712928, we conducted a DNA affinity capture assay as previously described [19]. Briefly, the 450 μL binding reactions consisted of 300 μg of pre-cleared, dialyzed MIN6 nuclear extract, 1X binding buffer, 50 ng/μL poly (dI-dC), and 40 pmol of biotin-labeled probe for either rs12712928 allele (same as EMSA probes) or a scrambled control. Binding reactions were incubated at room temperature for 30 min on a rotator, and then 100 μL of streptavidin-magnet Dynabeads were added to the reaction and incubated for an additional 20 minutes. Beads were washed and bound DNA-proteins were eluted in 1X reducing sample buffer. Proteins were separated on NuPAGE denaturing gel and allelic differences in protein bands was visualized with Coomassie G-250 staining. The UNC Michael Hooker Proteomics Center used a Sciex 5800 MALDI-TOF/TOF mass spectrometer to identify the proteins in the excised protein bands.

## Supporting information

S1 Text References for supplemental tables and figures. (DOCX)

S1 Fig Principal components analysis of allele frequency for CHNS (black) compared to other samples included in the International HapMap Project Phase 3. (A) All samples. (B) A subset of populations with East Asian ancestry.
(AI) Click here for additional data file.

S2 Fig Principal components analysis of allele frequency for the China Health and Nutrition Survey by province. Dots representing each subject are colored by the province in which they reside. Smaller plots show individuals from each province separately.
(AI) Click here for additional data file.

S3 Fig Principal components analysis of allele frequency for the China Health and Nutrition Survey by ethnicity. Dots representing each subject is colored by their self-reported ethnicity. Smaller plots show individuals for each ethnicity separately.
(AI) Click here for additional data file.

S4 Fig Flow chart of study design and sample sizes for association analyses. Of 8,403 subjects with genome-wide genotyping data after quality control, we analyzed 5,786 nondiabetic, fasting individuals for glucose and insulin; 6,943 nondiabetic individuals for HbA1c; and 5,731 non-first-degree relatives for type 2 diabetes.
(AI) Click here for additional data file.

S5 Fig Fasting glucose locus near *SIX3-SIX2* exhibits one association signal in the CHNS. (A) The purple diamond represents rs895636, the strongest associated variant in the initial unconditioned analysis of fasting glucose. (B) No additional association signals persist after conditioning on rs895636. Other variants are colored based on LD with the lead variant within 8,403 CHNS subjects.
(AI) Click here for additional data file.

S6 Fig Regional association plot of strongest fasting insulin association signal located near *CNTN6*. The purple diamond represents the lead variant, rs13078376, which exhibited the strongest evidence of association at the locus among 1000 Genomes Project Phase 3-imputed variants. Other variants are colored based on LD with the lead variant within 8,403 CHNS subjects.
(AI) Click here for additional data file.

S7 Fig Regional association plot of strongest HbA1c association signal in CHNS located near *FN3KRP*. The purple diamond represents the lead variant, rs9895455, which exhibited the strongest evidence of association at the locus among 1000 Genomes Project Phase 3-imputed variants. Other variants are colored based on LD with the lead variant within 8,403 CHNS subjects. Arrows indicate a previously reported variant.[10, 37].
(AI) Click here for additional data file.

S8 Fig Regional association plots of the strongest T2D loci identified in the CHNS. (A) *RTN4RRL1*, (B) *SOCS6*, (C) *ARID1B*. The purple diamond represents the lead variant, which exhibited the strongest evidence of association at the locus among 1000 Genomes Project Phase 3-imputed variants. Other variants are colored based on LD with the lead variant within 8,403 CHNS subjects. A nominal association (*P* = 0.04) has been detected between a variant at *ARID1B* and T2D.[43].
(AI) Click here for additional data file.

S9 Fig Chromatin marks in pancreatic islets in the intergenic region between *SIX3* and *SIX2*. UCSC Genome Browser (hg19) diagram showing the location of the lead fasting glucose variant, rs895636, and other candidate variants. *SIX3* is transcribed from left to right, while *SIX2* is transcribed from right to left. The region expanded below spans all candidate variants, including the lead GWAS variant, all variants in high LD (East Asian r^2^>0.80) with the lead GWAS variant, the lead European pancreatic islet eQTL variant (rs12712928), and all variants in high LD (European r^2^>0.80) with the lead eQTL variant. rs12712928, rs12712929, rs10168523, and rs748947 overlap regions of accessible chromatin in human pancreatic islets detected by H3K9ac ChIP-seq. Tested candidate regulatory elements (CREs) are represented by horizontal black rectangles in the lower figure.
(AI) Click here for additional data file.

S10 Fig rs748947 at the *SIX3-SIX2* locus does not exhibit allelic differences in transcriptional activity in MIN6 mouse insulinoma cells. rs748947 is a proxy of rs895636 and rs12712928, but does not appear to contribute to transcriptional activity differences at this locus.
(AI) Click here for additional data file.

S11 Fig Allele-specific binding of DNA-protein complexes in MIN6 nuclear extract for four candidate variants at SIX3-SIX2. Probes spanning each allele were incubated with MIN6 nuclear lysate and subjected to electromobility shift assay (EMSA). The only band indicative of a DNA-protein complex was observed for allele C of rs12712928.
(AI) Click here for additional data file.

S12 Fig (A) Allele-specific binding of DNA-protein complexes in MIN6 nuclear extract for four candidate variants at SIX3-SIX2. Probes spanning each allele were incubated with MIN6 nuclear lysate and subjected to electromobility shift assay (EMSA). The only band indicative of a DNA-protein complex was observed for allele C of rs12712928. (B) EMSA with biotin-labeled probes containing the C or G allele of rs12712928 show an allele specific band (arrow; lane 7 versus 2) that is competed away more effectively by 45-fold excess of unlabeled probe containing the C allele (lane 8) than the G allele (lane 9).
(PDF)

S13 Fig Chromatin marks in pancreatic islets spanning of the two fasting glucose-associated signals near *G6PC2*. (A) Signal 1. The variant with the strongest association with fasting glucose, rs34177044, and the only variant in high LD (r^2^>0.80), rs1402837, are shown. Variant rs1402837 is located in a region with evidence of regulatory potential at the *G6PC2* promoter, overlapping regions of accessible chromatin in human pancreatic islets detected by H3K4me3 ChIP-seq. Tested candidate regulatory elements (CREs) are represented by horizontal black rectangles. (B) Signal 2. Variant rs2232326 is situated in the last exon of *G6PC2* and encodes Ser324Pro. The two additional variants in high LD (r^2^≥0.80) with rs2232326 are located downstream within an intron of *ABCB11*. One variant, rs13387347, was previously reported to be associated with fasting glucose in East Asians.
(AI) Click here for additional data file.

S1 Table General characteristics of the CHNS subjects (A) and the number of type 2 diabetes cases identified using each of the case definition criteria (B). (XLSX)

S2 Table CHNS association results (*P*<0.05) for previously reported fasting glucose variants. (XLSX)

S3 Table Haplotype analysis of *G6PC2* in the China Health and Nutrition Survey with fasting glucose levels (n = 5,785). (XLSX)

S4 Table Fasting glucose association results in CHNS at known coding variants within *G6PC2*. (XLSX)

S5 Table CHNS association results (*P*<0.05) for previously reported fasting insulin variants. (XLSX)

S6 Table CHNS association results (*P*<0.05) for previously reported HbA1c variants. (XLSX)

S7 Table CHNS association results (*P*<0.05) for previously reported type 2 diabetes variants. (XLSX)

S8 Table Loci associated with type 2 diabetes in CHNS (*P*<5x10^-6^). (XLSX)

S9 Table eQTLs for SNPs with moderate to high LD (r^2^>0.80) with lead GWAS variants. (XLSX)

S10 Table Primer sequences for functional assays. (XLSX)

## References

[1] World Health Organization. Gobal Report on Diabetes. 2016.

[2] Ma RC, Chan JC. Type 2 diabetes in East Asians: similarities and differences with populations in Europe and the United States. Annals of the New York Academy of Sciences. 2013;1281:64–91. doi: 10.1111/nyas.12098 23551121 10.1111/nyas.12098 PMC3708105

[3] Fuchsberger C, Flannick J, Teslovich TM, Mahajan A, Agarwala V, Gaulton KJ, et al The genetic architecture of type 2 diabetes. Nature. 2016;536(7614):41–7. doi: 10.1038/nature18642 27398621 10.1038/nature18642 PMC5034897

[4] Mahajan A, Wessel J, Willems S, Zhao W, Robertson NR, Chu AY, et al Refining The Accuracy Of Validated Target Identification Through Coding Variant Fine-Mapping In Type 2 Diabetes. bioRxiv. 2017 doi: 10.1101/144410 10.1038/s41588-018-0084-1 PMC5898373 29632382

[5] Mohlke KL, Boehnke M. Recent advances in understanding the genetic architecture of type 2 diabetes. Human molecular genetics. 2015;24(R1):R85–92. doi: 10.1093/hmg/ddv264 26160912 10.1093/hmg/ddv264 PMC4572004

[6] Scott RA, Scott LJ, Magi R, Marullo L, Gaulton KJ, Kaakinen M, et al An Expanded Genome-Wide Association Study of Type 2 Diabetes in Europeans. Diabetes. 2017. 10.2337/db16-1253 PMC5652602 28566273

[7] Liu CT, Raghavan S, Maruthur N, Kabagambe EK, Hong J, Ng MC, et al Trans-ethnic Meta-analysis and Functional Annotation Illuminates the Genetic Architecture of Fasting Glucose and Insulin. American journal of human genetics. 2016;99(1):56–75. doi: 10.1016/j.ajhg.2016.05.006 27321945 10.1016/j.ajhg.2016.05.006 PMC5005440

[8] DIAGRAM, Asian Genetic Epidemiology Network Type 2 Diabetes C, South Asian Type 2 Diabetes C, Mexican American Type 2 Diabetes C, Type 2 Diabetes Genetic Exploration by Nex-generation sequencing in muylti-Ethnic Samples C, et al Genome-wide trans-ancestry meta-analysis provides insight into the genetic architecture of type 2 diabetes susceptibility. Nat Genet. 2014;46(3):234–44. doi: 10.1038/ng.2897 24509480 10.1038/ng.2897 PMC3969612

[9] Scott RA, Lagou V, Welch RP, Wheeler E, Montasser ME, Luan J, et al Large-scale association analyses identify new loci influencing glycemic traits and provide insight into the underlying biological pathways. Nature genetics. 2012;44(9):991–1005. doi: 10.1038/ng.2385 22885924 10.1038/ng.2385 PMC3433394

[10] Chen P, Takeuchi F, Lee JY, Li H, Wu JY, Liang J, et al Multiple nonglycemic genomic loci are newly associated with blood level of glycated hemoglobin in East Asians. Diabetes. 2014;63(7):2551–62. doi: 10.2337/db13-1815 24647736 10.2337/db13-1815 PMC4284402

[11] Hwang JY, Sim X, Wu Y, Liang J, Tabara Y, Hu C, et al Genome-Wide Association Meta-analysis Identifies Novel Variants Associated With Fasting Plasma Glucose in East Asians. Diabetes. 2015;64(1):291–8. doi: 10.2337/db14-0563 25187374 10.2337/db14-0563 PMC4274808

[12] Popkin BM, Du S, Zhai F, Zhang B. Cohort Profile: The China Health and Nutrition Survey—monitoring and understanding socio-economic and health change in China, 1989–2011. International journal of epidemiology. 2010;39(6):1435–40. doi: 10.1093/ije/dyp322 19887509 10.1093/ije/dyp322 PMC2992625

[13] Auton A, Brooks LD, Durbin RM, Garrison EP, Kang HM, Korbel JO, et al A global reference for human genetic variation. Nature. 2015;526(7571):68–74. doi: 10.1038/nature15393 26432245 10.1038/nature15393 PMC4750478

[14] Whitfield JB. Genetic insights into cardiometabolic risk factors. The Clinical biochemist Reviews / Australian Association of Clinical Biochemists. 2014;35(1):15–36. PMC3961996 24659834

[15] An integrated encyclopedia of DNA elements in the human genome. Nature. 2012;489(7414):57–74. doi: 10.1038/nature11247 22955616 10.1038/nature11247 PMC3439153

[16] Andersson R, Gebhard C, Miguel-Escalada I, Hoof I, Bornholdt J, Boyd M, et al An atlas of active enhancers across human cell types and tissues. Nature. 2014;507(7493):455–61. doi: 10.1038/nature12787 24670763 10.1038/nature12787 PMC5215096

[17] Bernstein BE, Stamatoyannopoulos JA, Costello JF, Ren B, Milosavljevic A, Meissner A, et al The NIH Roadmap Epigenomics Mapping Consortium. Nature biotechnology. 2010;28(10):1045–8. doi: 10.1038/nbt1010-1045 20944595 10.1038/nbt1010-1045 PMC3607281

[18] Schmitt AD, Hu M, Jung I, Xu Z, Qiu Y, Tan CL, et al A Compendium of Chromatin Contact Maps Reveals Spatially Active Regions in the Human Genome. Cell reports. 2016;17(8):2042–59. doi: 10.1016/j.celrep.2016.10.061 27851967 10.1016/j.celrep.2016.10.061 PMC5478386

[19] Fogarty MP, Cannon ME, Vadlamudi S, Gaulton KJ, Mohlke KL. Identification of a regulatory variant that binds FOXA1 and FOXA2 at the CDC123/CAMK1D type 2 diabetes GWAS locus. PLoS genetics. 2014;10(9):e1004633 doi: 10.1371/journal.pgen.1004633 25211022 10.1371/journal.pgen.1004633 PMC4161327

[20] Roman TS, Cannon ME, Vadlamudi S, Buchkovich ML, Wolford BN, Welch RP, et al A Type 2 Diabetes-Associated Functional Regulatory Variant in a Pancreatic Islet Enhancer at the ADCY5 Locus. Diabetes. 2017;66(9):2521–30. doi: 10.2337/db17-0464 28684635 10.2337/db17-0464 PMC5860374

[21] Mahajan A, Sim X, Ng HJ, Manning A, Rivas MA, Highland HM, et al Identification and functional characterization of G6PC2 coding variants influencing glycemic traits define an effector transcript at the G6PC2-ABCB11 locus. PLoS genetics. 2015;11(1):e1004876 doi: 10.1371/journal.pgen.1004876 25625282 10.1371/journal.pgen.1004876 PMC4307976

[22] Baerenwald DA, Bonnefond A, Bouatia-Naji N, Flemming BP, Umunakwe OC, Oeser JK, et al Multiple functional polymorphisms in the G6PC2 gene contribute to the association with higher fasting plasma glucose levels. Diabetologia. 2013;56(6):1306–16. doi: 10.1007/s00125-013-2875-3 23508304 10.1007/s00125-013-2875-3 PMC4106008

[23] Bouatia-Naji N, Bonnefond A, Baerenwald DA, Marchand M, Bugliani M, Marchetti P, et al Genetic and functional assessment of the role of the rs13431652-A and rs573225-A alleles in the G6PC2 promoter that are strongly associated with elevated fasting glucose levels. Diabetes. 2010;59(10):2662–71. doi: 10.2337/db10-0389 20622168 10.2337/db10-0389 PMC3279535

[24] Kang HM, Sul JH, Service SK, Zaitlen NA, Kong SY, Freimer NB, et al Variance component model to account for sample structure in genome-wide association studies. Nature genetics. 2010;42(4):348–54. doi: 10.1038/ng.548 20208533 10.1038/ng.548 PMC3092069

[25] Chen WM, Erdos MR, Jackson AU, Saxena R, Sanna S, Silver KD, et al Variations in the G6PC2/ABCB11 genomic region are associated with fasting glucose levels. The Journal of clinical investigation. 2008;118(7):2620–8. doi: 10.1172/JCI34566 18521185 10.1172/JCI34566 PMC2398737

[26] Dupuis J, Langenberg C, Prokopenko I, Saxena R, Soranzo N, Jackson AU, et al New genetic loci implicated in fasting glucose homeostasis and their impact on type 2 diabetes risk. Nature genetics. 2010;42(2):105–16. doi: 10.1038/ng.520 20081858 10.1038/ng.520 PMC3018764

[27] Kim YJ, Go MJ, Hu C, Hong CB, Kim YK, Lee JY, et al Large-scale genome-wide association studies in East Asians identify new genetic loci influencing metabolic traits. Nature genetics. 2011;43(10):990–5. doi: 10.1038/ng.939 21909109 10.1038/ng.939

[28] UniProt: the universal protein knowledgebase. Nucleic acids research. 2017;45(D1):D158–d69. doi: 10.1093/nar/gkw1099 27899622 10.1093/nar/gkw1099 PMC5210571

[29] Law EC, Wilman HR, Kelm S, Shi J, Deane CM. Examining the Conservation of Kinks in Alpha Helices. PloS one. 2016;11(6):e0157553 doi: 10.1371/journal.pone.0157553 27314675 10.1371/journal.pone.0157553 PMC4912094

[30] Ahn DH, Ozer HG, Hancioglu B, Lesinski GB, Timmers C, Bekaii-Saab T. Whole-exome tumor sequencing study in biliary cancer patients with a response to MEK inhibitors. Oncotarget. 2016;7(5):5306–12. doi: 10.18632/oncotarget.6632 26683364 10.18632/oncotarget.6632 PMC4868687

[31] Wessel J, Chu AY, Willems SM, Wang S, Yaghootkar H, Brody JA, et al Low-frequency and rare exome chip variants associate with fasting glucose and type 2 diabetes susceptibility. Nat Commun. 2015;6:5897 doi: 10.1038/ncomms6897 25631608 10.1038/ncomms6897 PMC4311266

[32] Xu S, Yin X, Li S, Jin W, Lou H, Yang L, et al Genomic dissection of population substructure of Han Chinese and its implication in association studies. American journal of human genetics. 2009;85(6):762–74. doi: 10.1016/j.ajhg.2009.10.015 19944404 10.1016/j.ajhg.2009.10.015 PMC2790582

[33] Manning AK, Hivert MF, Scott RA, Grimsby JL, Bouatia-Naji N, Chen H, et al A genome-wide approach accounting for body mass index identifies genetic variants influencing fasting glycemic traits and insulin resistance. Nature genetics. 2012;44(6):659–69. doi: 10.1038/ng.2274 22581228 10.1038/ng.2274 PMC3613127

[34] Comuzzie AG, Cole SA, Laston SL, Voruganti VS, Haack K, Gibbs RA, et al Novel genetic loci identified for the pathophysiology of childhood obesity in the Hispanic population. PloS one. 2012;7(12):e51954 doi: 10.1371/journal.pone.0051954 23251661 10.1371/journal.pone.0051954 PMC3522587

[35] Paterson AD, Waggott D, Boright AP, Hosseini SM, Shen E, Sylvestre MP, et al A genome-wide association study identifies a novel major locus for glycemic control in type 1 diabetes, as measured by both A1C and glucose. Diabetes. 2010;59(2):539–49. doi: 10.2337/db09-0653 19875614 10.2337/db09-0653 PMC2809960

[36] Soranzo N, Sanna S, Wheeler E, Gieger C, Radke D, Dupuis J, et al Common variants at 10 genomic loci influence hemoglobin A(1)(C) levels via glycemic and nonglycemic pathways. Diabetes. 2010;59(12):3229–39. doi: 10.2337/db10-0502 20858683 10.2337/db10-0502 PMC2992787

[37] Chen P, Ong RT, Tay WT, Sim X, Ali M, Xu H, et al A study assessing the association of glycated hemoglobin A1C (HbA1C) associated variants with HbA1C, chronic kidney disease and diabetic retinopathy in populations of Asian ancestry. PloS one. 2013;8(11):e79767 doi: 10.1371/journal.pone.0079767 24244560 10.1371/journal.pone.0079767 PMC3820602

[38] Walford GA, Gustafsson S, Rybin D, Stancakova A, Chen H, Liu CT, et al Genome-Wide Association Study of the Modified Stumvoll Insulin Sensitivity Index Identifies BCL2 and FAM19A2 as Novel Insulin Sensitivity Loci. Diabetes. 2016;65(10):3200–11. doi: 10.2337/db16-0199 27416945 10.2337/db16-0199 PMC5033262

[39] Mahajan A, Go MJ, Zhang W, Below JE, Gaulton KJ, Ferreira T, et al Genome-wide trans-ancestry meta-analysis provides insight into the genetic architecture of type 2 diabetes susceptibility. Nature genetics. 2014;46(3):234–44. doi: 10.1038/ng.2897 24509480 10.1038/ng.2897 PMC3969612

[40] Unoki H, Takahashi A, Kawaguchi T, Hara K, Horikoshi M, Andersen G, et al SNPs in KCNQ1 are associated with susceptibility to type 2 diabetes in East Asian and European populations. Nature genetics. 2008;40(9):1098–102. doi: 10.1038/ng.208 18711366 10.1038/ng.208

[41] Voight BF, Scott LJ, Steinthorsdottir V, Morris AP, Dina C, Welch RP, et al Twelve type 2 diabetes susceptibility loci identified through large-scale association analysis. Nature genetics. 2010;42(7):579–89. doi: 10.1038/ng.609 20581827 10.1038/ng.609 PMC3080658

[42] Willer CJ, Schmidt EM, Sengupta S, Peloso GM, Gustafsson S, Kanoni S, et al Discovery and refinement of loci associated with lipid levels. Nature genetics. 2013;45(11):1274–83. doi: 10.1038/ng.2797 24097068 10.1038/ng.2797 PMC3838666

[43] Gaulton KJ, Ferreira T, Lee Y, Raimondo A, Magi R, Reschen ME, et al Genetic fine mapping and genomic annotation defines causal mechanisms at type 2 diabetes susceptibility loci. Nature genetics. 2015;47(12):1415–25. doi: 10.1038/ng.3437 26551672 10.1038/ng.3437 PMC4666734

[44] The Genotype-Tissue Expression (GTEx) project. Nature genetics. 2013;45(6):580–5. doi: 10.1038/ng.2653 23715323 10.1038/ng.2653 PMC4010069

[45] Westra HJ, Peters MJ, Esko T, Yaghootkar H, Schurmann C, Kettunen J, et al Systematic identification of trans eQTLs as putative drivers of known disease associations. Nature genetics. 2013;45(10):1238–43. doi: 10.1038/ng.2756 24013639 10.1038/ng.2756 PMC3991562

[46] Fadista J, Vikman P, Laakso EO, Mollet IG, Esguerra JL, Taneera J, et al Global genomic and transcriptomic analysis of human pancreatic islets reveals novel genes influencing glucose metabolism. Proceedings of the National Academy of Sciences of the United States of America. 2014;111(38):13924–9. doi: 10.1073/pnas.1402665111 25201977 10.1073/pnas.1402665111 PMC4183326

[47] Varshney A, Scott LJ, Welch RP, Erdos MR, Chines PS, Narisu N, et al Genetic regulatory signatures underlying islet gene expression and type 2 diabetes. Proceedings of the National Academy of Sciences of the United States of America. 2017;114(9):2301–6. doi: 10.1073/pnas.1621192114 28193859 10.1073/pnas.1621192114 PMC5338551

[48] Mathelier A, Fornes O, Arenillas DJ, Chen CY, Denay G, Lee J, et al JASPAR 2016: a major expansion and update of the open-access database of transcription factor binding profiles. Nucleic acids research. 2016;44(D1):D110–5. doi: 10.1093/nar/gkv1176 26531826 10.1093/nar/gkv1176 PMC4702842

[49] Ogawa N, Biggin MD. High-throughput SELEX determination of DNA sequences bound by transcription factors in vitro. Methods in molecular biology (Clifton, NJ). 2012;786:51–63. 10.1007/978-1-61779-292-2_3 21938619

[50] Ward LD, Kellis M. HaploReg v4: systematic mining of putative causal variants, cell types, regulators and target genes for human complex traits and disease. Nucleic acids research. 2016;44(D1):D877–81. doi: 10.1093/nar/gkv1340 26657631 10.1093/nar/gkv1340 PMC4702929

[51] O’Brien RM. Moving on from GWAS: functional studies on the G6PC2 gene implicated in the regulation of fasting blood glucose. Current diabetes reports. 2013;13(6):768–77. doi: 10.1007/s11892-013-0422-8 24142592 10.1007/s11892-013-0422-8 PMC4041587

[52] Stranger BE, Stahl EA, Raj T. Progress and promise of genome-wide association studies for human complex trait genetics. Genetics. 2011;187(2):367–83. doi: 10.1534/genetics.110.120907 21115973 10.1534/genetics.110.120907 PMC3030483

[53] Ebert DH, Bischof LJ, Streeper RS, Chapman SC, Svitek CA, Goldman JK, et al Structure and promoter activity of an islet-specific glucose-6-phosphatase catalytic subunit-related gene. Diabetes. 1999;48(3):543–51. 10078554 10.2337/diabetes.48.3.543

[54] Bouatia-Naji N, Rocheleau G, Van Lommel L, Lemaire K, Schuit F, Cavalcanti-Proenca C, et al A polymorphism within the G6PC2 gene is associated with fasting plasma glucose levels. Science (New York, NY). 2008;320(5879):1085–8. 10.1126/science.1156849 18451265

[55] Horikoshi M, Mgi R, van de Bunt M, Surakka I, Sarin AP, Mahajan A, et al Discovery and Fine-Mapping of Glycaemic and Obesity-Related Trait Loci Using High-Density Imputation. PLoS genetics. 2015;11(7):e1005230 doi: 10.1371/journal.pgen.1005230 26132169 10.1371/journal.pgen.1005230 PMC4488845

[56] Pare G, Chasman DI, Parker AN, Nathan DM, Miletich JP, Zee RY, et al Novel association of HK1 with glycated hemoglobin in a non-diabetic population: a genome-wide evaluation of 14,618 participants in the Women’s Genome Health Study. PLoS genetics. 2008;4(12):e1000312 doi: 10.1371/journal.pgen.1000312 19096518 10.1371/journal.pgen.1000312 PMC2596965

[57] Prokopenko I, Langenberg C, Florez JC, Saxena R, Soranzo N, Thorleifsson G, et al Variants in MTNR1B influence fasting glucose levels. Nature genetics. 2009;41(1):77–81. doi: 10.1038/ng.290 19060907 10.1038/ng.290 PMC2682768

[58] Corradin O, Saiakhova A, Akhtar-Zaidi B, Myeroff L, Willis J, Cowper-Sal lari R, et al Combinatorial effects of multiple enhancer variants in linkage disequilibrium dictate levels of gene expression to confer susceptibility to common traits. Genome research. 2014;24(1):1–13. doi: 10.1101/gr.164079.113 24196873 10.1101/gr.164079.113 PMC3875850

[59] Roman TS, Marvelle AF, Fogarty MP, Vadlamudi S, Gonzalez AJ, Buchkovich ML, et al Multiple Hepatic Regulatory Variants at the GALNT2 GWAS Locus Associated with High-Density Lipoprotein Cholesterol. American journal of human genetics. 2015;97(6):801–15. doi: 10.1016/j.ajhg.2015.10.016 26637976 10.1016/j.ajhg.2015.10.016 PMC4678431

[60] Arda HE, Li L, Tsai J, Torre EA, Rosli Y, Peiris H, et al Age-Dependent Pancreatic Gene Regulation Reveals Mechanisms Governing Human beta Cell Function. Cell metabolism. 2016;23(5):909–20. doi: 10.1016/j.cmet.2016.04.002 27133132 10.1016/j.cmet.2016.04.002 PMC4864151

[61] Sonksen P, Sonksen J. Insulin: understanding its action in health and disease. British journal of anaesthesia. 2000;85(1):69–79. 10927996 10.1093/bja/85.1.69

[62] Gordon-Larsen P, Koehler E, Howard AG, Paynter L, Thompson AL, Adair LS, et al Eighteen year weight trajectories and metabolic markers of diabetes in modernising China. Diabetologia. 2014;57(9):1820–9. doi: 10.1007/s00125-014-3284-y 24891020 10.1007/s00125-014-3284-y PMC4119243

[63] Manichaikul A, Mychaleckyj JC, Rich SS, Daly K, Sale M, Chen WM. Robust relationship inference in genome-wide association studies. Bioinformatics (Oxford, England). 2010;26(22):2867–73. E 10.1093/bioinformatics/btq559 PMC3025716 20926424

[64] Sudmant PH, Rausch T, Gardner EJ, Handsaker RE, Abyzov A, Huddleston J, et al An integrated map of structural variation in 2,504 human genomes. Nature. 2015;526(7571):75–81. doi: 10.1038/nature15394 26432246 10.1038/nature15394 PMC4617611

[65] Das S, Forer L, Schonherr S, Sidore C, Locke AE, Kwong A, et al Next-generation genotype imputation service and methods. Nature genetics. 2016. 10.1038/ng.3656 PMC5157836 27571263

[66] Loh P-R, Palamara PF, Price A. Fast and accurate long-range phasing in a UK Biobank cohort. Nature genetics. 2016. 10.1038/ng.3571 PMC4925291 27270109

[67] Ma C, Blackwell T, Boehnke M, Scott LJ. Recommended joint and meta-analysis strategies for case-control association testing of single low-count variants. Genetic epidemiology. 2013;37(6):539–50. doi: 10.1002/gepi.21742 23788246 10.1002/gepi.21742 PMC4049324

[68] Surakka I, Horikoshi M, Magi R, Sarin AP, Mahajan A, Lagou V, et al The impact of low-frequency and rare variants on lipid levels. Nature genetics. 2015;47(6):589–97. doi: 10.1038/ng.3300 25961943 10.1038/ng.3300 PMC4757735

[69] Pruim RJ, Welch RP, Sanna S, Teslovich TM, Chines PS, Gliedt TP, et al LocusZoom: regional visualization of genome-wide association scan results. Bioinformatics (Oxford, England). 2010;26(18):2336–7. 10.1093/bioinformatics/btq419 PMC2935401 20634204

[70] T2D-GENES_Consortium, GoT2D_Consortium, DIAGRAM_Consortium. [cited 2017 February 1]. http://www.type2diabetesgenetics.org/home/portalHome.

[71] Schunkert H, Konig IR, Kathiresan S, Reilly MP, Assimes TL, Holm H, et al Large-scale association analysis identifies 13 new susceptibility loci for coronary artery disease. Nature genetics. 2011;43(4):333–8. doi: 10.1038/ng.784 21378990 10.1038/ng.784 PMC3119261

[72] Kottgen A, Pattaro C, Boger CA, Fuchsberger C, Olden M, Glazer NL, et al New loci associated with kidney function and chronic kidney disease. Nature genetics. 2010;42(5):376–84. doi: 10.1038/ng.568 20383146 10.1038/ng.568 PMC2997674

[73] Estrada K, Aukrust I, Bjorkhaug L, Burtt NP, Mercader JM, Garcia-Ortiz H, et al Association of a low-frequency variant in HNF1A with type 2 diabetes in a Latino population. JAMA: the journal of the American Medical Association. 2014;311(22):2305–14. doi: 10.1001/jama.2014.6511 24915262 10.1001/jama.2014.6511 PMC4425850

[74] Koesterer R. AMP-DCC Data Analysis Report, The Cardiology and Metabolic Patient Cohort (CAMP) Pfizer/MGH: Phase 1. 2017 February 7, 2017.

[75] Morris AP, Voight BF, Teslovich TM, Ferreira T, Segre AV, Steinthorsdottir V, et al Large-scale association analysis provides insights into the genetic architecture and pathophysiology of type 2 diabetes. Nature genetics. 2012;44(9):981–90. doi: 10.1038/ng.2383 22885922 10.1038/ng.2383 PMC3442244

[76] Locke AE, Kahali B, Berndt SI, Justice AE, Pers TH, Day FR, et al Genetic studies of body mass index yield new insights for obesity biology. Nature. 2015;518(7538):197–206. doi: 10.1038/nature14177 25673413 10.1038/nature14177 PMC4382211

[77] Shungin D, Winkler TW, Croteau-Chonka DC, Ferreira T, Locke AE, Magi R, et al New genetic loci link adipocyte and insulin biology to body fat distribution. Nature. 2015;518(7538):187–196. doi: 10.1038/nature14132 25673412 10.1038/nature14132 PMC4338562

[78] Saxena R, Hivert MF, Langenberg C, Tanaka T, Pankow JS, Vollenweider P, et al Genetic variation in GIPR influences the glucose and insulin responses to an oral glucose challenge. Nature genetics. 2010;42(2):142–8. doi: 10.1038/ng.521 20081857 10.1038/ng.521 PMC2922003

[79] Strawbridge RJ, Dupuis J, Prokopenko I, Barker A, Ahlqvist E, Rybin D, et al Genome-wide association identifies nine common variants associated with fasting proinsulin levels and provides new insights into the pathophysiology of type 2 diabetes. Diabetes. 2011;60(10):2624–34. doi: 10.2337/db11-0415 21873549 10.2337/db11-0415 PMC3178302

[80] Ehret GB, Ferreira T, Chasman DI, Jackson AU, Schmidt EM, Johnson T, et al The genetics of blood pressure regulation and its target organs from association studies in 342,415 individuals. Nature genetics. 2016;48(10):1171–84. doi: 10.1038/ng.3667 27618452 10.1038/ng.3667 PMC5042863

[81] Wu Y, Gao H, Li H, Tabara Y, Nakatochi M, Chiu YF, et al A meta-analysis of genome-wide association studies for adiponectin levels in East Asians identifies a novel locus near WDR11-FGFR2. Human molecular genetics. 2014;23(4):1108–19. doi: 10.1093/hmg/ddt488 24105470 10.1093/hmg/ddt488 PMC3900106

[82] Machiela MJ, Chanock SJ. LDlink: a web-based application for exploring population-specific haplotype structure and linking correlated alleles of possible functional variants. Bioinformatics (Oxford, England). 2015;31(21):3555–7. 10.1093/bioinformatics/btv402 PMC4626747 26139635

[83] Ernst J, Kheradpour P, Mikkelsen TS, Shoresh N, Ward LD, Epstein CB, et al Mapping and analysis of chromatin state dynamics in nine human cell types. Nature. 2011;473(7345):43–9. doi: 10.1038/nature09906 21441907 10.1038/nature09906 PMC3088773

[84] Kundaje A, Meuleman W, Ernst J, Bilenky M, Yen A, Heravi-Moussavi A, et al Integrative analysis of 111 reference human epigenomes. Nature. 2015;518(7539):317–30. doi: 10.1038/nature14248 25693563 10.1038/nature14248 PMC4530010

[85] Human genomics. The Genotype-Tissue Expression (GTEx) pilot analysis: multitissue gene regulation in humans. Science (New York, NY). 2015;348(6235):648–60. 10.1126/science.1262110 PMC4547484 25954001

[86] Schadt EE, Molony C, Chudin E, Hao K, Yang X, Lum PY, et al Mapping the genetic architecture of gene expression in human liver. PLoS biology. 2008;6(5):e107 doi: 10.1371/journal.pbio.0060107 18462017 10.1371/journal.pbio.0060107 PMC2365981

[87] Civelek M, Wu Y, Pan C, Raulerson CK, Ko A, He A, et al Genetic regulation of adipose gene expression and integration with GWAS loci and cardio-metabolic traits. American journal of human genetics. 2017;100(3):428–443. doi: 10.1016/j.ajhg.2017.01.027 28257690 10.1016/j.ajhg.2017.01.027 PMC5339333

[88] Miyazaki J, Araki K, Yamato E, Ikegami H, Asano T, Shibasaki Y, et al Establishment of a pancreatic beta cell line that retains glucose-inducible insulin secretion: special reference to expression of glucose transporter isoforms. Endocrinology. 1990;127(1):126–32. doi: 10.1210/endo-127-1-126 2163307 10.1210/endo-127-1-126

