# Supplementary material for: Identification and functional analysis of glycemic trait loci in the China Health and Nutrition Survey
Source: PLoS Genet. 2018 Apr 5;14(4):e1007275. doi: 10.1371/journal.pgen.1007275 (PMC5886383; doi:10.1371/journal.pgen.1007275)
Supplement: S1 Text — (DOCX) [file pgen.1007275.s001.docx]

**Supporting Information - References**

1. Hwang JY, Sim X, Wu Y, Liang J, Tabara Y, Hu C, et al. Genome-Wide Association Meta-analysis Identifies Novel Variants Associated With Fasting Plasma Glucose in East Asians. Diabetes. 2015 Jan;64(1):291-8.

2. Kim YJ, Go MJ, Hu C, Hong CB, Kim YK, Lee JY, et al. Large-scale genome-wide association studies in East Asians identify new genetic loci influencing metabolic traits. Nature genetics. 2011 Oct;43(10):990-5.

3. Dupuis J, Langenberg C, Prokopenko I, Saxena R, Soranzo N, Jackson AU, et al. New genetic loci implicated in fasting glucose homeostasis and their impact on type 2 diabetes risk. Nature genetics. 2010 Feb;42(2):105-16.

4. Chen WM, Erdos MR, Jackson AU, Saxena R, Sanna S, Silver KD, et al. Variations in the G6PC2/ABCB11 genomic region are associated with fasting glucose levels. The Journal of clinical investigation. 2008 Jul;118(7):2620-8.

5. Manning AK, Hivert MF, Scott RA, Grimsby JL, Bouatia-Naji N, Chen H, et al. A genome-wide approach accounting for body mass index identifies genetic variants influencing fasting glycemic traits and insulin resistance. Nature genetics. 2012 Jun;44(6):659-69.

6. Comuzzie AG, Cole SA, Laston SL, Voruganti VS, Haack K, Gibbs RA, et al. Novel genetic loci identified for the pathophysiology of childhood obesity in the Hispanic population. PloS one. 2012;7(12):e51954.

7. Soranzo N, Sanna S, Wheeler E, Gieger C, Radke D, Dupuis J, et al. Common variants at 10 genomic loci influence hemoglobin A(1)(C) levels via glycemic and nonglycemic pathways. Diabetes. 2010 Dec;59(12):3229-39.

8. Chen P, Takeuchi F, Lee JY, Li H, Wu JY, Liang J, et al. Multiple nonglycemic genomic loci are newly associated with blood level of glycated hemoglobin in East Asians. Diabetes. 2014 Jul;63(7):2551-62.

9. Mahajan A, Wessel J, Willems SM, Zhao W, Robertson NR, Chu AY, et al. Refining The Accuracy Of Validated Target Identification Through Coding Variant Fine-Mapping In Type 2 Diabetes. bioRxiv. 2017.

10. Scott RA, Scott LJ, Magi R, Marullo L, Gaulton KJ, Kaakinen M, et al. An Expanded Genome-Wide Association Study of Type 2 Diabetes in Europeans. Diabetes. 2017 May 31.

11. Kooner JS, Saleheen D, Sim X, Sehmi J, Zhang W, Frossard P, et al. Genome-wide association study in individuals of South Asian ancestry identifies six new type 2 diabetes susceptibility loci. Nature genetics. 2011 Aug 28;43(10):984-9.

12. Cho YS, Chen CH, Hu C, Long J, Ong RT, Sim X, et al. Meta-analysis of genome-wide association studies identifies eight new loci for type 2 diabetes in east Asians. Nature genetics. 2012 Jan;44(1):67-72.

13. Gaulton KJ, Ferreira T, Lee Y, Raimondo A, Magi R, Reschen ME, et al. Genetic fine mapping and genomic annotation defines causal mechanisms at type 2 diabetes susceptibility loci. Nature genetics. 2015 Dec;47(12):1415-25.

14. Morris AP, Voight BF, Teslovich TM, Ferreira T, Segre AV, Steinthorsdottir V, et al. Large-scale association analysis provides insights into the genetic architecture and pathophysiology of type 2 diabetes. Nature genetics. 2012 Sep;44(9):981-90.

15. Fuchsberger C, Flannick J, Teslovich TM, Mahajan A, Agarwala V, Gaulton KJ, et al. The genetic architecture of type 2 diabetes. Nature. 2016 Aug 4;536(7614):41-7.

16. Saxena R, Elbers CC, Guo Y, Peter I, Gaunt TR, Mega JL, et al. Large-scale gene-centric meta-analysis across 39 studies identifies type 2 diabetes loci. American journal of human genetics. 2012 Mar 9;90(3):410-25.

17. Perry JR, Voight BF, Yengo L, Amin N, Dupuis J, Ganser M, et al. Stratifying type 2 diabetes cases by BMI identifies genetic risk variants in LAMA1 and enrichment for risk variants in lean compared to obese cases. PLoS genetics. 2012 May;8(5):e1002741.

18. Li H, Gan W, Lu L, Dong X, Han X, Hu C, et al. A genome-wide association study identifies GRK5 and RASGRP1 as type 2 diabetes loci in Chinese Hans. Diabetes. 2013 Jan;62(1):291-8.

19. Hara K, Fujita H, Johnson TA, Yamauchi T, Yasuda K, Horikoshi M, et al. Genome-wide association study identifies three novel loci for type 2 diabetes. Human molecular genetics. 2014 Jan 1;23(1):239-46.

20. Tsai FJ, Yang CF, Chen CC, Chuang LM, Lu CH, Chang CT, et al. A genome-wide association study identifies susceptibility variants for type 2 diabetes in Han Chinese. PLoS genetics. 2010 Feb 19;6(2):e1000847.

21. Steinthorsdottir V, Thorleifsson G, Sulem P, Helgason H, Grarup N, Sigurdsson A, et al. Identification of low-frequency and rare sequence variants associated with elevated or reduced risk of type 2 diabetes. Nature genetics. 2014 Mar;46(3):294-8.

22. Voight BF, Scott LJ, Steinthorsdottir V, Morris AP, Dina C, Welch RP, et al. Twelve type 2 diabetes susceptibility loci identified through large-scale association analysis. Nature genetics. 2010 Jul;42(7):579-89.

23. Yamauchi T, Hara K, Maeda S, Yasuda K, Takahashi A, Horikoshi M, et al. A genome-wide association study in the Japanese population identifies susceptibility loci for type 2 diabetes at UBE2E2 and C2CD4A-C2CD4B. Nature genetics. 2010 Oct;42(10):864-8.

24. Zeggini E, Scott LJ, Saxena R, Voight BF, Marchini JL, Hu T, et al. Meta-analysis of genome-wide association data and large-scale replication identifies additional susceptibility loci for type 2 diabetes. Nature genetics. 2008 May;40(5):638-45.

25. Imamura M, Takahashi A, Yamauchi T, Hara K, Yasuda K, Grarup N, et al. Genome-wide association studies in the Japanese population identify seven novel loci for type 2 diabetes. Nat Commun. 2016 Jan 28;7:10531.

26. Zhao W, Rasheed A, Tikkanen E, Lee JJ, Butterworth AS, Howson JMM, et al. Identification of new susceptibility loci for type 2 diabetes and shared etiological pathways with coronary heart disease. Nature genetics. 2017 Oct;49(10):1450-7.

27. Tabassum R, Chauhan G, Dwivedi OP, Mahajan A, Jaiswal A, Kaur I, et al. Genome-wide association study for type 2 diabetes in Indians identifies a new susceptibility locus at 2q21. Diabetes. 2013 Mar;62(3):977-86.

28. Palmer ND, McDonough CW, Hicks PJ, Roh BH, Wing MR, An SS, et al. A genome-wide association search for type 2 diabetes genes in African Americans. PloS one. 2012;7(1):e29202.

29. Qi L, Cornelis MC, Kraft P, Stanya KJ, Linda Kao WH, Pankow JS, et al. Genetic variants at 2q24 are associated with susceptibility to type 2 diabetes. Human molecular genetics. 2010 Jul 1;19(13):2706-15.

30. Rung J, Cauchi S, Albrechtsen A, Shen L, Rocheleau G, Cavalcanti-Proenca C, et al. Genetic variant near IRS1 is associated with type 2 diabetes, insulin resistance and hyperinsulinemia. Nature genetics. 2009 Oct;41(10):1110-5.

31. Shu XO, Long J, Cai Q, Qi L, Xiang YB, Cho YS, et al. Identification of new genetic risk variants for type 2 diabetes. PLoS genetics. 2010 Sep 16;6(9):e1001127.

32. Moltke I, Grarup N, Jorgensen ME, Bjerregaard P, Treebak JT, Fumagalli M, et al. A common Greenlandic TBC1D4 variant confers muscle insulin resistance and type 2 diabetes. Nature. 2014 Aug 14;512(7513):190-3.

33. Saxena R, Saleheen D, Been LF, Garavito ML, Braun T, Bjonnes A, et al. Genome-wide association study identifies a novel locus contributing to type 2 diabetes susceptibility in Sikhs of Punjabi origin from India. Diabetes. 2013 May;62(5):1746-55.

34. T2D-GENES_Consortium, GoT2D_Consortium, DIAGRAM_Consortium. [cited 2017 February 1]. Available from: <http://www.type2diabetesgenetics.org/home/portalHome>.

35. Parra EJ, Below JE, Krithika S, Valladares A, Barta JL, Cox NJ, et al. Genome-wide association study of type 2 diabetes in a sample from Mexico City and a meta-analysis of a Mexican-American sample from Starr County, Texas. Diabetologia. 2011 Aug;54(8):2038-46.

36. Unoki H, Takahashi A, Kawaguchi T, Hara K, Horikoshi M, Andersen G, et al. SNPs in KCNQ1 are associated with susceptibility to type 2 diabetes in East Asian and European populations. Nature genetics. 2008 Sep;40(9):1098-102.

37. Yasuda K, Miyake K, Horikawa Y, Hara K, Osawa H, Furuta H, et al. Variants in KCNQ1 are associated with susceptibility to type 2 diabetes mellitus. Nature genetics. 2008 Sep;40(9):1092-7.

38. Koesterer R. AMP-DCC Data Analysis Report, The Cardiology and Metabolic Patient Cohort (CAMP) Pfizer/MGH: Phase 1. 2017 February 7, 2017.

39. Takeuchi F, Serizawa M, Yamamoto K, Fujisawa T, Nakashima E, Ohnaka K, et al. Confirmation of multiple risk Loci and genetic impacts by a genome-wide association study of type 2 diabetes in the Japanese population. Diabetes. 2009 Jul;58(7):1690-9.

40. Ng MC, Shriner D, Chen BH, Li J, Chen WM, Guo X, et al. Meta-analysis of genome-wide association studies in African Americans provides insights into the genetic architecture of type 2 diabetes. PLoS genetics. 2014 Aug;10(8):e1004517.

41. Imamura M, Maeda S, Yamauchi T, Hara K, Yasuda K, Morizono T, et al. A single-nucleotide polymorphism in ANK1 is associated with susceptibility to type 2 diabetes in Japanese populations. Human molecular genetics. 2012 Jul 1;21(13):3042-9.

42. Ma RC, Chan JC. Type 2 diabetes in East Asians: similarities and differences with populations in Europe and the United States. Annals of the New York Academy of Sciences. 2013 Apr;1281:64-91.
