# Supplementary figures and images for: Identification and functional analysis of glycemic trait loci in the China Health and Nutrition Survey
Source: PLoS Genet. 2018 Apr 5;14(4):e1007275. doi: 10.1371/journal.pgen.1007275 (PMC5886383; doi:10.1371/journal.pgen.1007275)

Figure S12A

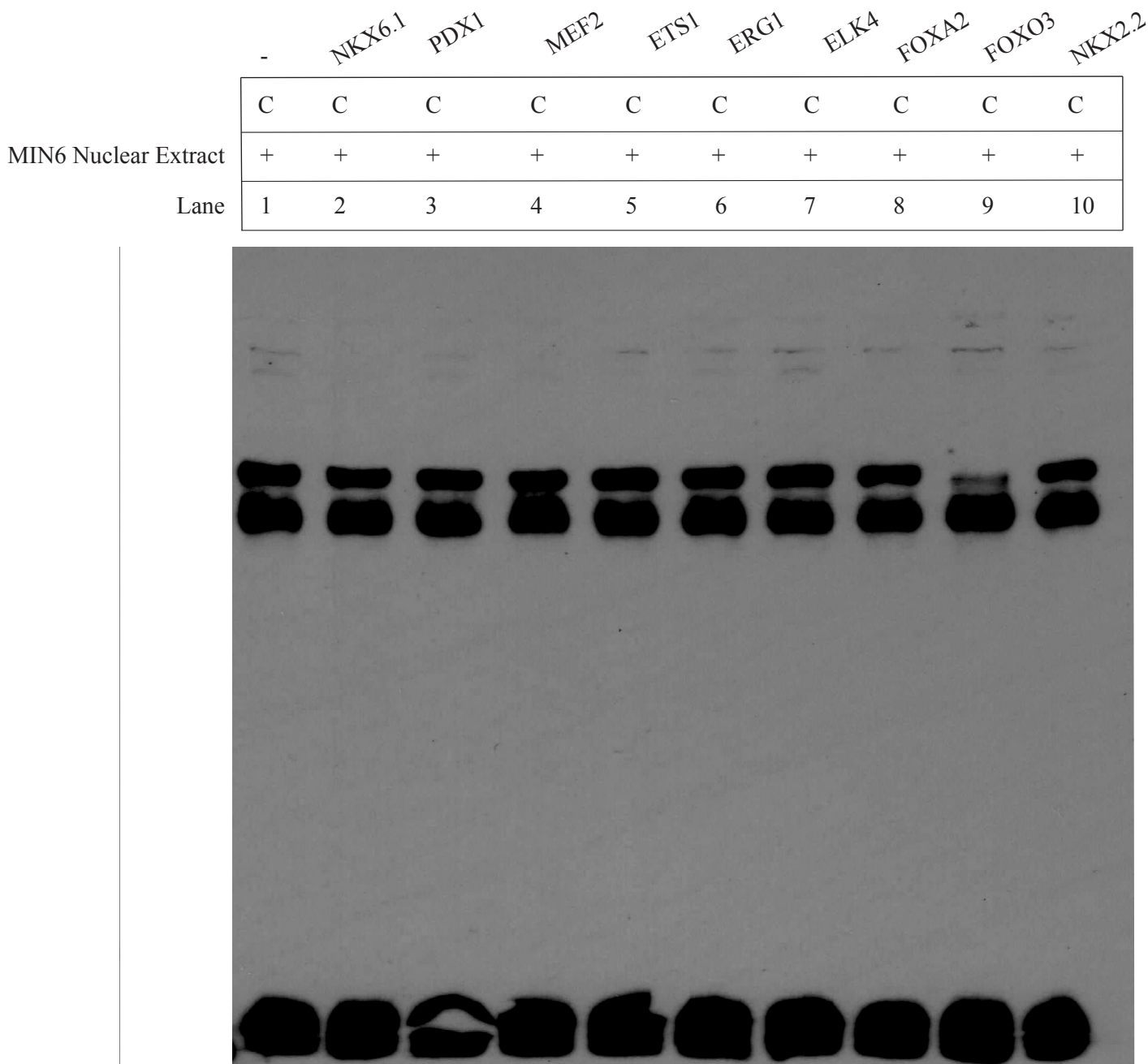

Figure S12B

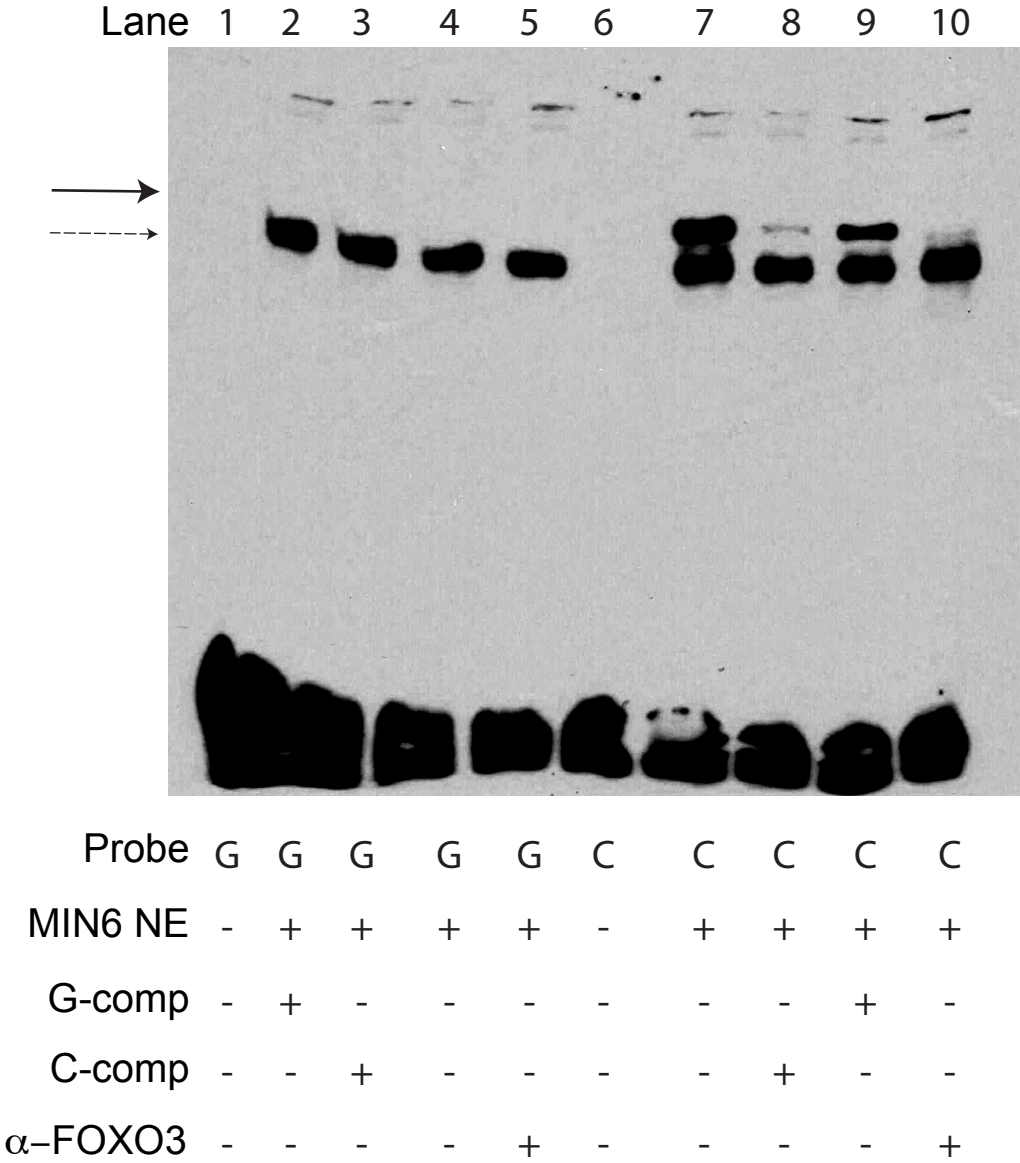

Supplement: S12 Fig — (A) Allele-specific binding of DNA-protein complexes in MIN6 nuclear extract for four candidate variants at SIX3-SIX2. Probes spanning each allele were incubated with MIN6 nuclear lysate and subjected to electromobility shift assay (EMSA). The only band indicative of a DNA-protein complex was observed for allele C of rs12712928. (B) EMSA with biotin-labeled probes containing the C or G allele of rs12712928 show an allele specific band (arrow; lane 7 versus 2) that is competed away more effectively by 45-fold excess of unlabeled probe containing the C allele (lane 8) than the G allele (lane 9). (PDF) [file pgen.1007275.s013.pdf]
